# Public platform with 39,472 exome control samples enables association studies without genotype sharing

**DOI:** 10.1038/s41588-023-01637-y

**Published:** 2024-01-10

**Authors:** Mykyta Artomov, Alexander A. Loboda, Maxim N. Artyomov, Mark J. Daly

**Affiliations:** 1 Institute for Genomic Medicine, Nationwide Children’s Hospital, Columbus, OH USA; 2 Department of Pediatrics, The Ohio State University College of Medicine, Columbus, OH USA; 3 Analytic and Translational Genetics Unit, Massachusetts General Hospital, Boston, MA USA; 4 Broad Institute, Cambridge, MA USA; 5 ITMO University, St. Petersburg, Russia; 6 Almazov National Medical Research Center, St. Petersburg, Russia; 7 Department of Immunology and Pathology, Washington University in St. Louis, St. Louis, MO USA; 8 Institute for Molecular Medicine Finland, Helsinki, Finland

**Keywords:** Genetics, Biological techniques

## Abstract

Acquiring a sufficiently powered cohort of control samples matched to a case sample can be time-consuming or, in some cases, impossible. Accordingly, an ability to leverage genetic data from control samples that were already collected elsewhere could dramatically improve power in genetic association studies. Sharing of control samples can pose significant challenges, since most human genetic data are subject to strict sharing regulations. Here, using the properties of singular value decomposition and subsampling algorithm, we developed a method allowing selection of the best-matching controls in an external pool of samples compliant with personal data protection and eliminating the need for genotype sharing. We provide access to a library of 39,472 exome sequencing controls at http://dnascore.net enabling association studies for case cohorts lacking control subjects. Using this approach, control sets can be selected from this online library with a prespecified matching accuracy, ensuring well-calibrated association analysis for both rare and common variants.

## Abstract (web summary)

A new method allows selection of matched controls from an external pool of samples without genotype sharing. This method has been implemented in an online repository containing 39,472 exome sequencing controls that can be used for association analyses.

## Main

The success of genetic association studies critically depends not only on the collection of case samples but also on the quality and size of the collected control samples to ensure that discovered associations are phenotype driven. Control cohort subjects are selected in a way that minimizes technical and ancestral biases between case and control cohorts. While technical biases are well controlled by using the same sequencing technology and data processing standards for case and control cohorts, the common genetic background of cases and control subjects has to be actively enforced during the study design stage. This can be achieved either by recruiting study-specific controls or by selecting appropriate controls from already published studies within databases like dbGAP^1^. The latter, however, is often very challenging from both technical and regulatory perspectives. Such databases typically consist of multiple relatively small cohorts (hundreds of individuals), and each one requires separate access and extensive post-processing to combine them into a single dataset before any statistical analysis. Even then, only a subset of such samples could serve as appropriate controls for a given case cohort, as they would have to undergo rigorous selection based on ancestry matching. Each of these steps requires explicit genotype sharing, which serves as one of the major obstacles to the efficient utilization of public control pools^2^.

The theoretical possibility of association studies without sharing individual-level data has been discussed widely: UNICORN^3^ proposed to create a potential control repository for GWAS/genotyping array studies with precomputed base ancestry space such that both case and control data could be projected on it for further matching. Yet, practical implementation of this concept at scale has not emerged. Several methods, for example, TRAPD^4^, CoCoRV^5^ and Summix^6^, proposed computational solutions to utilize publicly available allele frequencies and genotype counts from gnomAD as a pool of controls. Such approaches are often limited only to the analysis of rare disease, often caused by de novo variants, where population structure does not play a critical role in signal detection^7^ and therefore ancestry matching between cases and controls (TRAPD). Alternatively, the ancestry matching is limited to the predefined major continental groups defined in gnomAD, substantially limiting the possibility of including admixed or fine-scale ancestry cohorts into the analysis (CoCoRV and Summix). Collaborative Spanish Variant Server framework offered a user with an ability to select samples that should be included in the control subset based on their phenotypes, yet this solution was limited only to copy-number variation and Spanish population, without the mechanism to perform ancestry matching^8^. GLADDB solution, proposed recently for sharing genetic data for Latin-American cohorts, potentially could be viewed as a step toward practical data sharing; however, this solution implies open individual-level data sharing, such as individual coordinates of the samples in the principal component space. As such, this solution could only be used for cohorts that already exist in the public domain and are already approved for open individual-level data sharing^9^.

Currently, several alternatives to direct data sharing are available. For example, large-scale analytical initiatives, such as AllofUS^10^, UK Biobank^11^ and FinnGen^12^, provide secure cloud environments that permit direct interaction with individual-level data for authorized users. While being highly effective, such solutions limit the utility of the data for external usage and usually require a thorough multistep process of user identification. Conclusively, a practical, fully secure framework for case–control association studies without individual-level data sharing is a highly desirable, though yet unachieved, goal.

In this Technical Report, we consider a situation in which a researcher has assembled a case cohort and is interested in performing an association study using allele frequencies estimated in a well-matched control cohort from a common public repository. To achieve this, we used insights from singular value decomposition (SVD) applications^13^ and developed a methodology for selecting background-matched control sets without explicit genotype or individual-level data sharing. We evaluated our approach in a series of large-scale genetic data analyses and implemented an online portal (SVD-based Control Repository (SCoRe), www.dnascore.net) that contains 39,472 controls. Our implementation selects optimal control subjects and provides summary genotype counts for the selected control set, such that the investigator can locally perform an association study. SCoRe allows researchers worldwide to select the most optimal controls in a manner compatible with data sharing regulations, thus enabling massive improvement in studies’ statistical power.

## Results

### Overview of the framework for control selection without genotype sharing

In case of shared genotypes, selection of ancestrally matched control cohort is conducted through analyzing coordinates of samples in the shared principal component space. Individual coordinates, however, cannot be shared, and the need for determining relative positioning of case and control cohorts in the same coordinate basis is the main challenge that our method aims to address.

We assume a situation where genetic data for a cohort of cases are directly available for analysis but lack control subjects. First, we use SVD to decrease the dimensionality of the centered genotype matrix for the control pool. Commonly, the first vector-columns of the left-singular vector matrix represent the directions of the maximal variability. We assume that the control pool has a broader population structure than a cohort of cases, and thus, first vector-columns of the left-singular vector matrix derived from the pool of controls, will be used as a basis for the case-control matching process. This basis can be shared to the local site, and the projections of case genotypes can be obtained. Next, we apply a similar SVD operation to the matrix of case coordinates in the control basis. The resulting left singular value matrix would represent the directions of the greatest variance in the genotypes of the case cohort within the basis of controls. Unlike individual-level coordinates, sharing such information from the case cohort with a remote control repository is permitted without restrictions since it lacks any individual-level data. Importantly, this information is sufficient to parametrize the Gaussian-like distribution of the case subjects’ projections in the space spanned by the left-singular vectors of control genotype matrix. This can be accomplished by computing the covariance matrix and the mean value using the maximum likelihood estimator. Common standards for data quality filtration, outlier detection and missing genotypes imputation will apply (‘Shareable data generation’ section in Methods and Supplementary Figs. 1–3). We also require sharing the summary allele counts that will further be used in evaluation of the control selection quality.

On a remote server, we set up the process of subsampling the control pool in such a way that the distribution parameters of the proposed set of controls fit the best to the target distribution of the case samples. To measure the similarity between the proposed set of controls and a case cohort, we use the Baringhaus–Henze–Epps–Pulley statistic, defined as the difference between characteristic functions of the target distribution and the sample distribution weighted on a Gaussian kernel^14^. The process of selection of an optimal subset of controls is then formulated as an optimization problem aimed to minimize the BHEP statistic, which is solved with a simulated annealing approach^15^ (‘Control selection using a remote server’ section in Methods). Further, the summary allele counts are used to compute the association test statistic for variants that were used for control matching, and genomic inflation is assessed. The largest control set delivering the genomic inflation below a user-defined threshold is therefore selected as optimal, and summary allele counts are returned to the user.

**Fig. 1:**
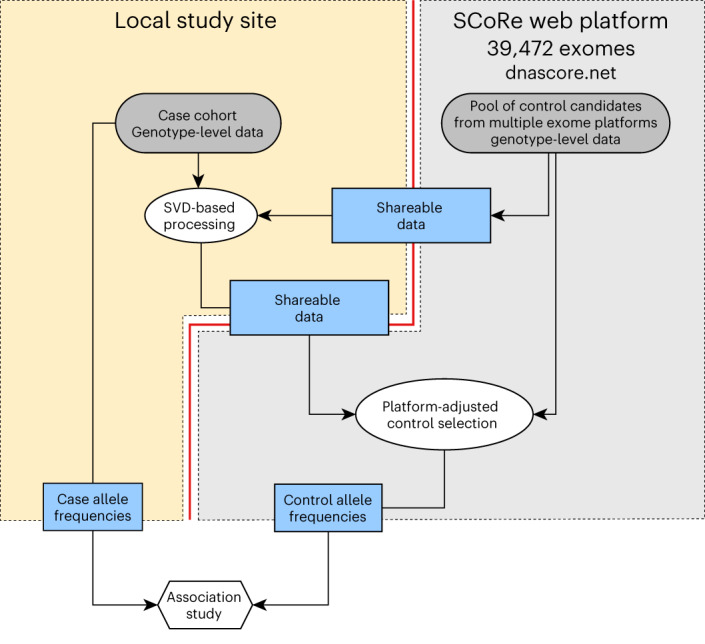
Scheme of an association study without genotype sharing. Individual-level genotype data are subject to data sharing restrictions. SVD-based processing creates anonymous data describing variation in case genotypes without storing individual data that could be shared with no restrictions. Remote server with a pool of controls selects a set of control genotype variation matching cases, estimates allele frequency for sites to be used for association study and delivers results to the user.

Using this approach, we created SCoRe, a public platform with 39,472 exome sequencing controls (Supplementary Tables 1 and 2), and a complementary R package, SVDFunctions (Fig. 1). Further, we describe extensive process of evaluation of performance and robustness of the approach using large-scale genetic data from multiple technical platforms, and major continental and fine-scale ancestry groups.

### Global populations dataset of 16,532 exomes

#### Cross-validation in a random set of cases

**Fig. 2:**
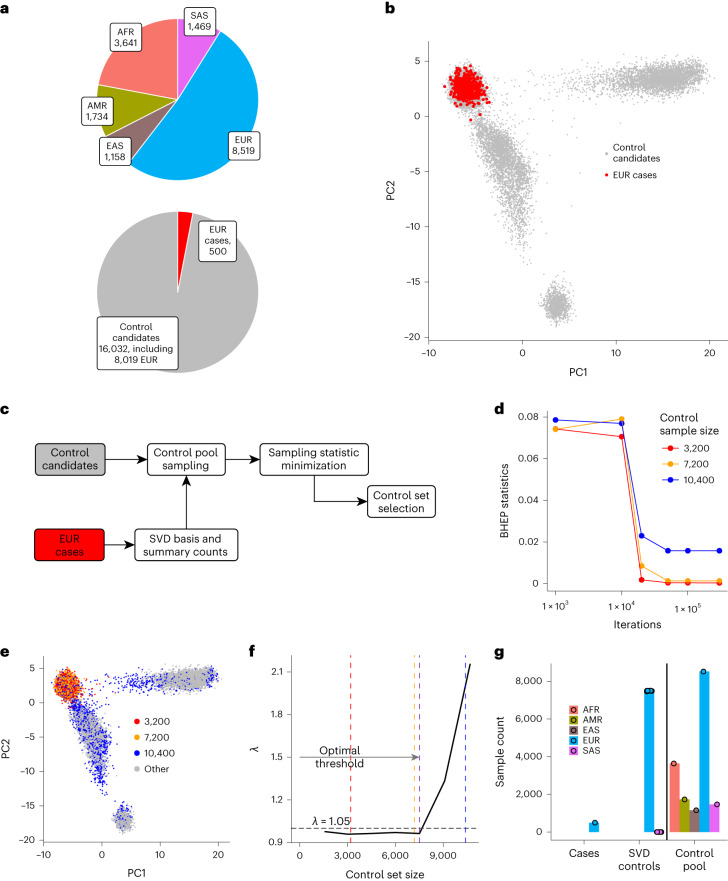
Case study 1: simulated case–control study with 16,532 exomes dataset. **a**, Breakdown of continental ancestries present in the dataset and case–control study setup: random 500 European samples are selected as ‘cases’, and the rest of the data is tested as prospective controls. **b**, Conventional PCA showing European samples selected as case group. **c**, Scheme of data handling simulating association study without genotype sharing. **d**, Sampling statistic minimization for control candidate sets of different size for a specific case cohort. **e**, Conventional PCA shows that greater size of the control candidate sets to be sampled deteriorates their quality, as can be seen by inclusion of samples of nontarget ancestry. **f**, Optimal size of the control pool for a given case cohort is selected to deliver the largest set of samples with *λ*_GC_ < 1.05. **g**, Matching experiment summary results over ten random sets of European cases (error bars represent standard error; center of the error bars represents mean). AFR, African and African American; AMR, Latin American; EAS, East Asian; EUR, European and European-American; SAS, South Asian.

To illustrate our approach, we first assembled the dataset of exome sequences representing major global populations by downloading dbGAP studies suitable for usage as control subjects and permitted for general research (Fig. 2a and Supplementary Table 1). All individual studies were combined into a single dataset through joint variant calling. The raw dataset was subjected to a quality filtering workflow (Supplementary Fig. 4) yielding a final data freeze of 16,532 samples. All samples in the aggregated dataset were sequenced with Agilent exome capture at the Broad Institute. Common coding linkage disequillibrium pruned (LD-pruned) variants were selected for constructing the genotype matrix. To simulate an association study, we randomly divided the dataset into 500 European ‘cases’ and a ‘control candidate pool’ of the remaining 16,032 samples, which included 8,019 Europeans (Fig. 2a–c).

Following the control selection protocol, the projections of case vectors on the basis of the control pool were computed and fitted with Gaussian distribution. Next, the parameters of the distribution and summary genotype counts of the case cohort were transmitted to the control pool.

The BHEP statistic was then optimized for each size of the control candidate set (from 100 to 16,000 with a step of 500 samples, Fig. 2d). Increasing the size of the control candidate set naturally leads to deteriorating control quality, as can be seen by inclusion of samples of nontarget ancestry (Fig. 2e). The quality of the control candidate cohort can be evaluated through genomic control for linear regression test statistics (*λ*)^16^, estimated using summary genotype counts from cases and control candidate sets (Fig. 2f).

To select the optimal size of the control set, we computed *λ* for each size of the control candidate pool, and the largest control set with *λ* < 1.05 became an optimal control set (Fig. 2f). Figure 2g illustrates the results of running 100 random simulations for a European ‘case’ cohort and the reliability of the selection of the control set.

We performed a parameter sensitivity analysis to illustrate that our method works consistently well for major continental populations, keeps the false positive rate low and benefits from the larger case cohort sizes (Supplementary Fig. 5).

#### Fine-scale ancestry matching in an independent dataset

In the above experiment setting, the case cohort was randomly drawn from the pool of European samples present in the control dataset. Expectedly, such an approach returns the case cohort with distribution of European subpopulations similar to the one observed in Europeans of the control pool. Moreover, the case and control cohorts were part of the same joint variant calling process, which may have eliminated the technical biases.

We illustrated the method’s robustness to analytic pipeline differences and alterations in composition of subpopulations in case cohort compared to the control repository. We used a 1000 Genomes^17^ dataset of OMNI microarray genotyping as a source for case cohorts from each fine-scale ancestry (only directly genotyped variants were used). Importantly, the case and control datasets represent different genotype discovery technologies and downstream data processing. Therefore, such experimental design entirely eliminates the potential artificial advantages of similarities in data processing between case and control cohorts.

We used 18 local subpopulations from 1000 Genomes as simulated case cohorts and performed case–control selection without genotype sharing using the Global Populations dataset as a control pool. All but five local populations were adequately matched to control sets (Supplementary Fig. 6). We observed that, for the subpopulations for which our method was unable to return control sets, there were no samples of the corresponding ancestry in the pool of controls (Supplementary Fig. 7). Therefore, our method is robust with respect to the absence of joint calling and does not return control cohorts for samples of local subpopulations that do not have representation in the pool of controls (‘Fine-scale ancestry matching in independent datasets’ section in Supplementary Note).

#### Matching cases with internal structure of subpopulations

We used the same 1000 Genomes data to keep only individuals of European descent as a case cohort, consisting of a composition of fine-scale ancestries—Finnish (FIN), Utah residents with Northern and Western European ancestry (CEU), Iberian populations in Spain (IBS), Toscani in Italia (TSI) and British in England and Scotland (GBR)—which is different from the fine-scale ancestry composition in the Global Populations dataset (‘Selecting controls for case cohorts with internal structure of subpopulations’ section in Supplementary Note and Supplementary Fig. 8a). First, we identified the clusters within the case cohort that corresponded to the southern Europe, western Europe and Finnish populations (Supplementary Fig. 8b–e). The shareable data for a clustered case cohort were then used to select controls, resulting in control cohorts adequately selected for each cluster independently (Supplementary Fig. 8f–i; note, there are only 45 Finnish samples in the Global Population dataset, resulting in a small control cohort for the Finnish case cluster).

Conclusively, parameter sensitivity tests suggest that noise, artifacts generated by data processing pipelines, and limitations of the exome sequencing data commonly observed for joint case–control datasets are well tolerated by our algorithm.

### Nordic Dataset of 22,940 exomes

#### Cross-validation in a random set of cases

**Fig. 3:**
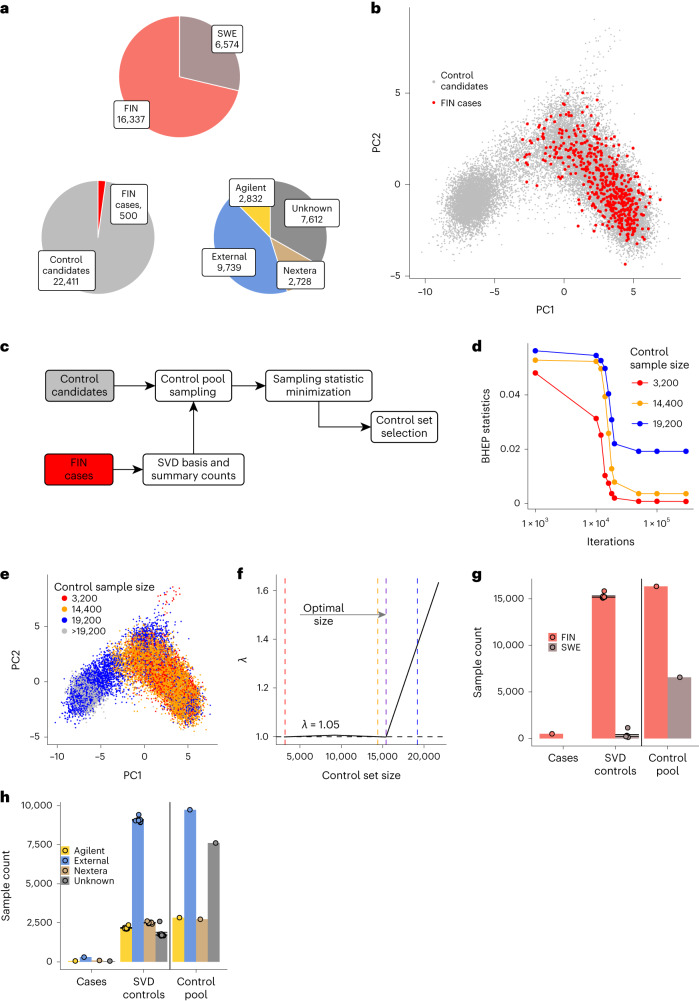
Case study 2: simulated case–control study with 22,911 exomes dataset. **a**, Breakdown of fine-scale ancestries, different exome platforms present in the dataset, and case–control study setup: random 500 Finnish samples are selected as ‘cases’ and the rest of the data is tested as prospective controls. **b**, Conventional PCA showing Finnish samples selected as a case group. **c**, Scheme of data handling simulating association study without genotype sharing. **d**, Sampling statistic minimization for control candidate sets of different size for the selected case cohort. **e**, Conventional PCA shows that greater size of the control candidate sets to be sampled deteriorates their quality, as can be seen by inclusion of samples of nontarget ancestry. **f**, Optimal size of the control pool for a given case cohort is selected to deliver the largest set of samples with *λ*_GC_ < 1.05. **g**, Matching experiment summary results over ten random sets of Finnish cases (error bars represent standard error; center of the error bars represents mean). **h**, Samples from multiple sequencing platforms are successfully selected as control group in ten random case sets (error bars represent standard error; center of the error bars represents mean). FIN, Finnish ancestry; SWE, Swedish ancestry.

The Global Populations dataset is relatively uniform in technical processing and does not reflect the full complexity of technical biases that may exist in sequencing data. Thus, we next considered a separate, nonoverlapping dataset of 22,940 exomes of individuals from northern Europe (Sweden and Finland), sequenced on multiple exome capture kits and coming from multiple sequencing centers (Fig. 3a and Supplementary Table 2). The dataset was subjected to quality filtering, and 11,286 common autosomal coding LD-pruned variants were selected for genotype matrix construction. Five-hundred Finnish samples were selected as a ‘case’ group, and the control selection procedure was performed without genotype sharing as described above (Fig. 3b–f). Figure 3g shows that our approach robustly selects controls of Finnish ancestry, even given the limited ability of exome variants to distinguish fine-scale European ancestries.

#### Investigation of effects of exome sequencing platforms

Selection of control samples independent of exome capture kits (Fig. 3h) suggested that differences in sequencing platforms might not interfere with the control selection process. The genotype principal component analysis (PCA) space does not immediately reflect the presence of multiple sequencing platforms in the data (Supplementary Fig. 9). We further confirmed this by selecting a case cohort from Nordic Dataset consisting only of Finnish samples sequenced using Nextera capture and successfully selecting a set of controls from a control pool lacking Nextera samples. Importantly, our algorithm delivers a control dataset with both common and rare variants matched (Supplementary Fig. 10).

Furthermore, we eliminated the benefit of joint variant calling present in the experiment above, and used 45 Finnish samples from the Global Populations dataset (Agilent exome capture) as a case cohort and a control pool from Nordic Dataset from which we eliminated the Agilent sequencing platform samples. The algorithm selected 1,708 Finnish samples from Nordic Dataset, indicating robustness to the study origin and independence of data processing (Supplementary Fig. 11).

A possible reason for this platform insensitivity could be the way standard data quality check routines are designed. One of the conventional data curation steps is a variant call rate filter, which keeps only those variants that have nonmissing genotype in at least 90% of samples (Supplementary Fig. 4). Such a filter eliminates variants that have notable variation in call rates across different exome sequencing platforms. The absence of call rate variation could protect from observing platform-biased allele frequencies and therefore would yield noninflated association statistics, enabling efficient control selection.

**Fig. 4:**
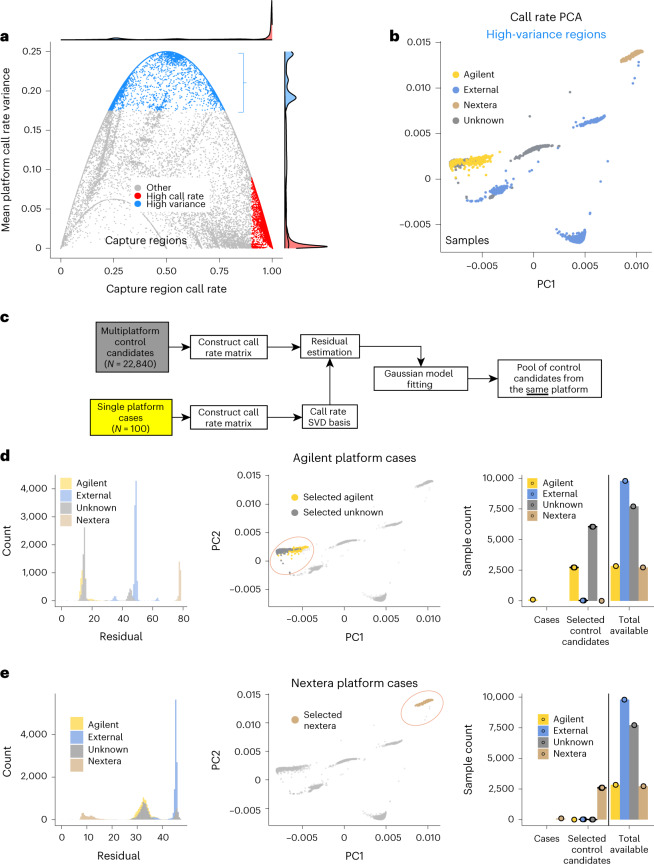
Evaluation of platform bias effect and platform selection. **a**, Mean call rate across all samples (*X* axis) and variance of mean call rates for samples coming from the same platform (*Y* axis) were estimated for each capture interval. Each point represents a capture interval. Marginal distributions show that the majority of the regions have high call rate (>0.9) and low variance between platforms (highlighted in red). Regions with high call rate variance are highlighted in blue. **b**, PCA performed on a matrix of mean call rates per individual (columns) per capture region (rows); each point represents an individual sample. Points are colored with respect to reported capture used for sequencing. **c**, Workflow scheme for selecting control candidates for ancestry matching from the most appropriate platform. **d**,**e**, Workflow was tested for Agilent (**d**) and Nextera (**e**) platform ‘cases’. Left: residual norm distributions. Middle: call rate-based PCA separates sequencing platforms with every point representing a sample. Highlighted are control candidates selected as a result of workflow execution for Agilent and Nextera ‘case’ cohort, respectively. Right: results of control candidates selection for ten random ‘case’ cohorts from each sequencing platform (error bars represent standard error).

To better understand this source of variability, we investigated the call rate properties of exome sequencing platforms to find genomic regions that best describe their differences (Fig. 4a). We estimated mean call rate for every genomic interval within samples coming from the same platform and further computed variance of these values for every interval. In fact, regions with high call rate (>0.9) have low variance in call rates between platforms, explaining the lack of sensitivity to the platform during the control selection.

Hence, standard data quality check protocol with variant call rate filter (even within data coming from a single platform) should be sufficient to overcome possible concerns about sequencing platform bias in SVD-based control selection.

### Matching samples based on sequencing platform

In certain contexts, however, it might be important to have a set of controls matched not only by ancestry but also by sequencing platform. Matching of platforms can be performed on the basis of regions with high variance in call rate between platforms. We used 11,407 high-variance (>0.175) genomic regions to construct a call rate matrix with rows representing genomic intervals and columns representing samples. Each entry in such a matrix is a mean call rate per sample per genomic interval. The PCA of this matrix efficiently separates the samples by sequencing platform^18^ (Fig. 4b), similar to the genotype-based PCA that separates the samples by ancestry. Thus, in the settings when individual-level genotype sharing is allowed, the genotype matrix of high variance genomic regions can be used to explicitly match samples between two cohorts to the same platform.

In situations where genotype sharing is not possible, one can utilize a simple SVD-based approach, similar to the algorithm commonly used in pattern recognition problems^13^. Specifically, such an algorithm would locally generate SVD of cases call rate matrix *С* *=* USV′, extract appropriate number of left singular vectors ${U}_{1..K}=\{{U}^{(i)}{|}\forall {i}{\in }{[}{1}..{K}{]}\}$ and transmit it to central repository where control candidates (${\bf v}_{i}$) will be ranked by similarity of the call rate pattern evaluated by estimation of residual vector norm ${r}={||}({I}-{U}_{1..K}^{T}){\bf v}_{i}{||}$ (‘Selection of control samples from the specific sequencing platform’ section in Supplementary Note and Fig. 4c). Residual vector norms form Gaussian-like distributed clusters that correspond to individual platforms (Fig. 4d,e). We used the Mclust^19^ library to fit optimal Gaussian models to observed distribution of residual vector norms and identify homogeneous call rate clusters of prospective controls.

We evaluated this approach by running 100 random selections of ‘case’ group from Agilent and Nextera platforms and selecting control candidates using call rate matrix SVD-based approach. Figure 4d,e illustrates that this approach leads to a robust selection of samples that match the ‘case’ group platform. Interestingly, in the case of Agilent capture ‘cases’, a subset of selected control samples originates from an unknown sequencing platform (Fig. 4d). Upon closer examination, samples from unknown capture are found within the Agilent cluster on call rate-based PCA, suggesting that for this set of samples Agilent capture was used, though labeled as ‘unknown’. We performed further statistical evaluation (sensitivity to number of transmitted vectors, case cohort siz and so on) of this algorithm, confirming robustness of such approach (Supplementary Fig. 12).

Therefore, one can select ancestry-matched control sets from fixed exome platforms without individual genotype sharing using call rate-based SVD matching followed by genotype-based control selection.

### Case studies

**Fig. 5:**
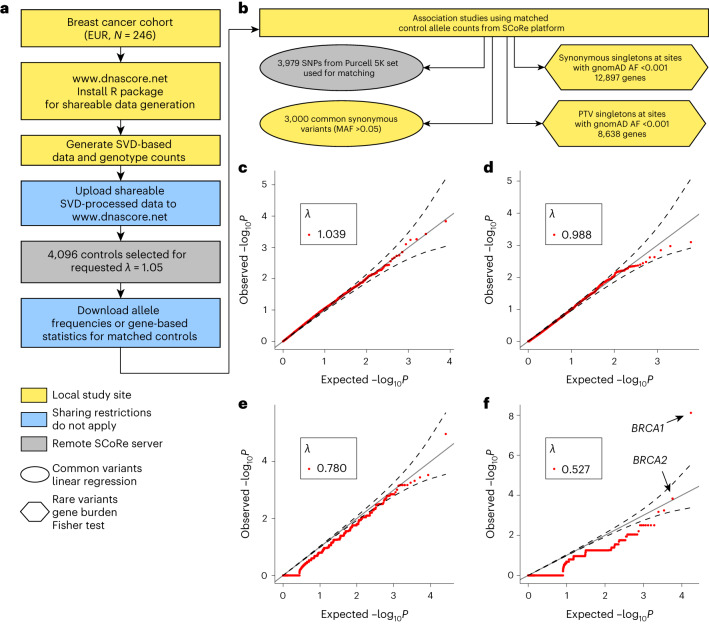
Breast cancer samples (*N* = 244) matched to controls (*N* = 4,096) using SCoRe web service. **a**, Workflow scheme for association study without genotype sharing. **b**, Control sets selected using different user-defined matching quality thresholds (*λ*). **c**, QQ plots for linear regression association statistics for every selection threshold on DNA variants used for matching. **d**, QQ plot for linear regression association statistics using summary genotypes counts from optimal control dataset (*λ* < 1.05) on common synonymous DNA variant. **e**, QQ plot for Fisher’s exact test association statistics using summary gene burden statistics for synonymous singletons on DNA variants with allele frequency <1 × 10^−3^ or not present in gnomAD. The solid line represents the diagonal, and the dashed lines indicate the 95% confidence interval (two-sided Fisher’s exact test). Raw, unadjusted *P* values are reported. **f**, QQ plot for Fisher’s exact test association statistics using summary gene burden statistics for protein-truncating singletons on DNA variants with allele frequency <1 × 10^−3^ or not present in gnomAD. The solid line represents the diagonal, and the dashed lines indicate the 95% confidence interval (two-sided Fisher’s exact test). Raw, unadjusted *P* values are reported. EUR, European and European-American ancestry; AF, allele frequency; SNP, single nucleotide polymorphism.

Next, we explored whether SCoRe can accurately select controls for rare variant gene-based association studies. We analyzed exome sequencing data for a cohort of patients with early-onset breast cancer (dbGAP: phs000822.v1.p1, Supplementary Note): 244 nonrelated cases matching quality standards were used for analysis^20^. Genotype matrix and summary genotype counts of cases were constructed for 3,979 LD-pruned DNA variants passing quality control, and shareable data were generated and uploaded to the SCoRe server (Fig. 5a). SCoRE yielded 4,096 controls matched to the case cohort with *λ* = 1.04. First, to confirm that selected controls are matched not only on variants that were used for shareable data generation, we used a list of common synonymous variants (that were not used for matching) in the case cohort and a list of genes with at least one singleton variant in cases (at sites with frequency less than 1/10,000 in gnomAD) for ultrarare burden calibration. For each list we downloaded summary control data from SCoRe and locally performed association analyses (linear regression for common variants and gene burden Fisher test for rare variants; Fig. 5b). As a result, both common (*λ* = 0.965) and rare (*λ* = 0.987) background variations were well calibrated (Fig. 5c–e), providing confidence in the further association study.

We submitted a list of 8,268 genes with at least one singleton protein-truncating variant (PTV) carrier (at sites with frequency less than 1/10,000 in gnomAD) in cases (Fig. 5b) to SCoRe to obtain summary PTV counts by gene from the control cohort. Local association study with Fisher’s test (Bonferroni-corrected significance threshold 0.05/8,268 = 6.05 × 10^−6^) was performed ‘re-discovering’ *BRCA1* and *BRCA2* as breast cancer susceptibility genes (Fig. 5f).

We performed the rare-variant association tests with different thresholds for the minor allele frequency to illustrate that selected controls are well matched to the case cohort in a wide range of minor allele frequencies (Supplementary Fig. 13).

Next, we performed a conventional case–control matching procedure with shared genotypes and obtained 2,786 controls (Supplementary Fig. 14) and compared the statistical power of the association study using the SCoRe and conventional approach. We estimated statistical power using simulations for Fisher’s exact test (statmod R package)^21^ with multiple odds ratios and allele frequencies for SCoRe test (Supplementary Fig. 15), implying 244 case cohort and single-batch matched control cohort of 4,096 samples. We observed that using SCoRe for control selection virtually saturated statistical power, making the size of the case cohort the limiting factor, which is the optimal scenario for local, clinical-based case-focused cohorts. Moreover, in the case of treating the case cohort as a single cluster, the control set selected by SCoRe is larger than could be obtained in case of genotype sharing following the common matching techniques.

Furthermore, two additional case studies were performed using African-American-derived populations to illustrate the practical utility of our approach for the underrepresented populations, for which SCoRe could become a step in solving data access limitations and inequalities. First, we performed pan-cancer analysis of the African-American subset of The Cancer Genome Atlas cohort (*N* = 471). After successfully matching 496 controls using the SCoRe server, we validated the matching using association testing for common and rare synonymous variants. Interestingly, both African-American and admixed African population clusters were successfully matched with a set of controls, indicating that our method is working for admixed populations. Analysis of rare PTV variants also appeared well calibrated, with *PRIM2* being the top associated gene. Interestingly, *PRIM2* was previously found to have the highest mutation rate in prostate tumors in patients of African-American descent^22^ (Supplementary Figs. 16 and 17).

Finally, a panel sequencing of 2,482 genes in a cohort of 130 African Americans diagnosed with focal segmental glomerulosclerosis (FSGS) was analyzed using SCoRe. Despite only 724 LD-pruned variants available for analyzing the population structure in the sequencing panel, SCoRe returned 700 controls with *λ* = 1.00. Common variant analysis resulted in replication of the known, G1 variant association in *APOL1* (ref. ^23^) (Supplementary Fig. 18).

Conclusively, SCoRe could be successfully used for case cohorts lacking control subjects to perform both common and rare-variant association studies, while returning meaningful and well-calibrated results. The SCoRe framework includes quality and data feature filters enabling its integration into local workflows for multiple types of data analysis, including meta-analysis and rare-variant association studies.

## Discussion

Local cohorts assembled at hospitals as part of clinical screening procedures or genetic counseling often have very modestly sized (or none at all) matched control sets and often have sensibly stringent data-sharing regulation. Especially for rare Mendelian phenotypes, the assembly of a well-powered case–control cohort is impeded by low disease prevalence. Despite the potential availability of control sets through public repositories, great effort should be put into processing case and control datasets jointly before even preliminary results of an association study could emerge. Practically, this often becomes infeasible for small cohort studies limited by data access or computational power. Large case–control datasets such as those assembled by international consortia (for example, ExAC/gnomAD, Psychiatrics Genomics Consortium and IBD Genetics Consortium) often provide access to summary allele frequencies and dataset quality properties. However, such resources represent data freezes of summary allele counts, which cannot be used as a one-size-fits-all model for association studies. The inability to subset the data and prioritize specific samples within these control pools as a best fit for a given case cohort is a major obstacle in using these resources for case–control association studies.

We provide a pool of 39,472 exome sequences and a tool enabling rapid selection of matched control sets without genotype sharing that ultimately outputs allele frequency statistics required for performing association tests. Importantly, all the other preparatory steps are the same for shared genotypes and should be performed as usual. In such settings, minimal effort is required from the user side to obtain all the information needed for an association study, thereby facilitating future discovery of associated genes and DNA variants. One potential limitation of our approach is the usage of prespecified set of variants of good quality (that is, low variance across platforms in call rate) for selecting the control set. Although this set of LD-pruned common variants (MAF >0.01) provides sufficient resolution for continental ancestry matching, it may not be sufficient for optimal fine-scale ancestry matching. Therefore, additional checks are needed for certain types of analysis. For example, our case studies suggest that rare variant gene burden in synonymous variants is well matched as a result of using our platform; however, we recommend that this be routinely checked by the user on a case-by-case basis. With respect to the admixed populations, our method is agnostic of the case population and works better if the distribution of cases can be shaped into a Gaussian form in the PCA space. This is usually not true for admixed populations, but we provide an explicit solution for ‘normalizing’ the case cohort. Yet, improving the efficiency of our methodology for all possible analyses in complex admixed cohorts would require additional research.

Although ancestry-associated matching is important, it would still be impossible to perform an association study without controlling for data quality differences and potential technical artifacts. The SCoRe design is fully compatible with other approaches focused on eliminating technical artifacts, such as platform-biased allele frequency estimates and coverage differences. Methods such as iECAT^24^ and ProxECAT^25^ provide a computational framework to control for technical differences in allele frequencies and could potentially complement SCoRe platform in cases when technical bias cannot be eliminated by selecting controls from appropriate sequencing platform or using common quality check standards.

Hundreds of thousands of samples have been subjected to exome or genome sequencing so far in the world. However, all these data exist in isolated pieces with highly regulated access, which limits the scope of population genetic studies. Here we provide a repository of the software codes for SCoRe implementation, so that it could readily be set up by large independent data holders—national biobank initiatives and international disease consortia to let the community benefit from large-scale genetic resources. This is also critically important for advancing genetic association studies in situations when explicit data sharing is not permitted or very challenging in international settings, thus potentially providing insights into rare sample collections that were not available so far. Finally, the approach developed in this work charts a path to creating a unified central repository that would encompass all studies published in dbGAP and make it accessible to association studies run in any design and cohort without compromising individual data security.

## Methods

There were no project-specific data generated, and therefore, no approval from the ethical committee was required. Public data utilized in our study have obtained relevant approvals, as indicated in corresponding referenced publications.

### Shareable data generation

We assume the situation when exome sequencing data for a cohort of cases are directly available for analysis, but lack the control subjects. We will describe the procedure of selecting the matched control subjects for a case cohort using a remote server storing sequencing data for a pool of controls without genotype sharing.

Both cohorts could be represented by their genotype matrices constructed using common (MAF >0.01) autosomal, LD-pruned variants, that are routinely used for the PCA in the scenario of shared genotypes^26,27^. The rows of the genotype matrix represent variants (*n*), and the columns represent samples (*m*). Each genotype entry is encoded by the number of alternative alleles —0, 1 or 2 (‘Genotype matrix generation’ section in Supplementary Note).

We provide a simple method to create such a genotype matrix directly from the VCF file and perform the required genotype (DP and GQ), variant and sample quality filtration. Infrequent missing values in the genotype matrices are imputed at the time of construction with a random forest model trained on the genotypes of the neighboring variants (‘Genotype imputation’ section in Supplementary Note, and Supplementary Fig. 1). Imputation is needed solely for the following steps of linear algebra operations that require complete data. The imputed genotypes are not used for computing association test statistics. Therefore, we keep two matrices to store the imputed and nonimputed data. First is the numeric genotype matrix with imputed values; second is the genotype matrix with missing values used for genotype counts calculation for association tests.

Let *G* be a genotype matrix of the control pool and **μ** be a vector of mean values of rows of *G*. Next, we will use the SVD for dimensionality reduction. Let *I*_*n*×*m*_ be a matrix of size *n* × *m* with ones on the main diagonal and zeros for off-diagonal elements. SVD can be applied to the centered matrix:${\overline{G}}={G}-{\bf \mu} {I}_{n\times m}={{\mathrm{USV}}^\prime}$. Commonly, singular values, $S_{ii}$, are put in descending order, so the first vectors-columns from $U$ represent the directions of the maximal variability. We assume that the control pool has a broader population structure than a cohort of cases, and, thus, it will be used as a base for the case–control matching process.

Similarly to the association tests that use first several principal components as covariates in the test model, we use the first ten vectors ${U}_{10}=\{{U}^{(i)}{|}\forall {i}{\in }{[}{1}..{10}{]}\}$ from $U$ to represent a set of orthogonal directions of maximal variance in projected data and encodes a population structure of the pool of controls. The matrix ${U}_{10}$ represents coordinates of the vectors forming the same orthonormal basis as occurring in PCA and does not have any individual level information. Therefore, it could be unrestrictedly shared. We will use this as a space to unite the case and control projections.

The control selection process is initiated by sharing the vectors ${\bf \mu}$ and matrix ${U}_{10}$ of control pool to a local machine that has the genotypes of a case cohort. It is critical that genotype matrices for case and control pool cohorts have the same variants; therefore, we provide a recommended set of autosomal common LD-pruned variants that was used to build the basis in the pool of controls (‘Genotype matrix generation’ section in Supplementary Note).

Next, the genotype matrix of cases, *H*, is built using the same variants as in the pool of controls. We run the test to check if all variants from the control pool matrix are present in the case dataset. Otherwise, only the subset of variants from the control pool that are found in cases is used, which requires an additional step of inversion of the reduced ${U}_{10}$ to combine case and control projections in the same basis (‘Harmonizing the genotype matrices of cases and control pool’ section in Supplementary Note).

The projections of the columns of *H* (representing case individuals) are then obtained as ${U}_{10}$: ${P}={U}_{10}^{T}({H}-{\bf \mu})$. This information is stored locally and is not shared.

Similarly to the server side, we apply the SVD on the client side. Let ${\bf{\mu}}_{\mathbf{P}}$ be row means of the matrix $P$. The centered matrix ${\overline{P}}$ of projections is obtained and decomposed: ${\overline{P}}={P}-{\bf{\mu}}_{\mathbf{P}}{I}_{n\times 10}={U}_{P}{S}_{P}{V}_{P}^{T}$. Here we drop the matrix ${V}_{P}^{T}$ containing individual-level information about cases and will use the rest to generate the shareable information for the case cohort.

We use a Gaussian model to describe the population structure in the case cohort. The Gaussian model is parametrized using the maximum likelihood estimator—mean value ${\bf{\mu }}_{\mathbf{P}}$ and covariance matrix $K$, which are the same as sample mean and sample covariance. The ${\bf{\mu }}_{\mathbf{P}}$ could be directly computed from the summary genotype counts (unrestrictedly shareable data) in the case genotype matrix. The covariance matrix could be obtained as $K={U}_{P}{S}_{P}{S}_{P}^{T}{U}_{P}^{T}$. Therefore, to describe the population structure in a case cohort, it is sufficient to share the summary genotype counts and the matrix ${U}_{P}{S}_{P}$, representing the coordinates of directions of the largest variance in case cohort projected to the basis of control pool. Both variables do not have any data that could be linked to a single individual. As a result, they can be unrestrictedly shared. We provide a convenient functionality to generate a single YAML file with a structured shareable data (‘Shareable data structure—YAML file’ section in Supplementary Note^28^).

### Control selection using a remote server

On a remote server, we set up the process of subsampling the control pool in such a way that the distribution parameters of the proposed set of controls fit the best to the proposed target distribution of the case samples.

The similarity between the proposed set of controls and a case cohort is measured with the BHEP statistic—a difference between characteristic functions of the target distribution and the sample distribution weighted on a Gaussian kernel^14^. Selection of an optimal subset of controls is formulated as an optimization problem aimed to minimize the BHEP statistic, which is solved with a simulated annealing approach^15^ (‘Subsampling control set using simulated annealing’ section in Supplementary Note).

The underlying distribution of BHEP statistic depends on the number of elements in a sample (the size of the proposed set of controls). Therefore, in general cases, a solution minimizing BHEP statistic could be obtained for each prespecified size of the control set, but subsamples of different sizes could not be compared between themselves on the basis of the BHEP statistic.

To determine the optimal size of the matched control dataset, we perform the sampling for each possible number of samples. For each subset size, we then compute the fitness of the prospective control set through an association study, involving only variants from the case genotype matrix $H$. If genomic control (*λ*) of the resulting test statistic is within a predefined soft threshold (*λ* ≤ 1.05 by default), this subset becomes a candidate solution. Among all candidate solutions, the one with the largest number of controls is selected for the return to a user. If no candidate solutions were found within the soft threshold, then the subset with the smallest *λ* is chosen for return. We define a hard threshold (*λ* ≤ 1.3 by default) as a limit for genomic inflation after which the control selection process is considered unreliable. If among all sampled control subsets none satisfies the hard threshold criterion, then no controls will be returned to the user.

Additional important parts of the framework include steps that are similar to the analysis conducted on shared genotypes, such as detection of outliers in case cohort and detection of multiple ancestry clusters in the case cohort and subsequent control selection approach using the remote server (Supplementary Figs. 2 and 3, and ‘Outlier detection and cohort PCA normalization; multiple ancestry clusters’ section in Supplementary Note).

### SVDFunctions R package and SCoRe online platform

We implemented described algorithm in R package^29^ ‘SVDFunctions’^30,31^ that provides all routines necessary for usage of our approach both as a user and for setting up an independent control pool repository.

We also provide access to SCoRe that enables association studies without genotype sharing (Fig. 1). Genotypes for the 39,744 potential control samples that are allowed for general research use are stored on the SCoRe server, accepting shareable SVD-processed data from local case clients and performing control cohort selection. Further, genotype frequencies for the variants of interest are computed in the selected control dataset and made available to a client to run a full-scale association test. Unlike individual-level data, such summary statistic sharing from most consented resources is routinely allowed^17,18,32^.

### Pool of controls exome sequencing data

We assembled two large-scale exome datasets that were used to illustrate the performance of the method and are provided as a public pool of controls for the remote association studies through the control server (‘Exome dataset details’ section in Supplementary Note).

#### Global Populations dataset

Whole exome libraries were prepared using the Whole Exome Agilent 1.1 RefSeq plus 3 boosters capture kit and protocol, automated on the Agilent Bravo and Hamilton Starlet. Libraries were then prepared for sequencing using a modified version of the manufacturer’s suggested protocol, automated on the Agilent Bravo and Hamilton Starlet, followed by sequencing on the Illumina HiSeq 2000. We used an aggregated set of samples consented for joint variant calling resulting in 37,607 samples and then created a subset of 16,532 samples approved for sharing through the SCoRe platform (Supplementary Table 1).

#### Nordic Dataset

The dataset was assembled from samples coming from multiple studies (Supplementary Table 2). Agilent 1.1 RefSeq plus 3 boosters, Illumina Nextera and several unknown exome capture kits. Some samples, labeled as ‘external’, were sequenced at a different sequencing facility; otherwise, all samples for both datasets were sequenced at the Broad Institute and aligned on the reference genome with BWA^33^ and the best-practices GATK/Picard Pipeline, followed by joint variant calling with all samples processed as a single batch using GATK v 3.1-144 Haplotype Caller^34–36^. The variant- and individual-based quality check protocol is available at ‘Exome sequencing data QC’ section in Supplementary Note. Variant effect predictor was used for variant annotation^37^. Missing genotypes for SVD were imputed using a custom random forest predictor (‘Shareable data generation’ section in Methods, and ‘Genotype imputation’ section in Supplementary Note).

#### Case studies

A dataset of the early onset breast cancer cohort^20^ was used as an illustration of the method’s performance in the actual association study. It is available through dbGAP (phs000822.v1.p1, ‘Case study. Breast cancer association study using the SCoRe platform’ section Supplementary Note).

The utility of the method for the non-European case studies was illustrated using African-American pan-cancer cohort from The Cancer Genome Atlas^38^ (dbGAP: phs000178.v11.p8) and African-American subgroup of the FSGS cohort^39^ (‘Case study. African-American cohorts’ section in Supplementary Note).

#### SVDFunctions R package and SCoRe online platform

The SCoRe platform returns summary genotype counts for a selected set of well-matched controls, along with the QQ plot and the corresponding genomic inflation factor *λ*. SCoRe allows users to control the quality of control selection by setting a maximal *λ* threshold—a stricter threshold will result in a smaller but more accurately selected control dataset.

In addition to ancestry matching, successful association studies require that genotype filters are also matched in case and control cohorts with respect to the sequencing depth and genotype qualities (DP and GQ fields in VCF format). Also, it is possible to indicate a variant call rate that should be used as a filter when calculating genomic inflation factor. We provide an option to specify individual genotype filters for data returned to a user from selected controls. This way only genotypes consistent with provided options will contribute to outputs.

Finally, in case of rare variant gene-based association studies, another parameter needs to be matched between case and control cohorts: minor allele frequency filters enable restriction of gene statistics aggregation to rare variants based on frequencies of alleles in selected control cohort or in gnomAD data^18^. This ensures that allele frequency thresholds are the same in case and control cohorts and variants are aggregated into gene burden tests based on the same principles. Furthermore, calibration of rare variation is also critically important for gene-based tests. This is usually done by evaluating gene-based association statistics aggregated from rare synonymous variants and ensuring the absence of inflation. Therefore, we provide the ability to output statistics in accord with Variant Effect Predictor (VEP)^37^ variant annotations to restrict output to synonymous, missense or protein-truncating variants. Importantly, when using minor allele frequency thresholds for the control data output, it is vital to use compatible threshold values between case and control cohorts. For example, if a case cohort includes only 50 samples, the minimal achievable MAF is 0.01 and, therefore, the control cohort could not be subjected to a smaller threshold for MAF to avoid artificial bias creation (‘Control data access and control set genotype counts generation, SCoRe server design’ section in Supplementary Note).

### Reporting summary

Further information on research design is available in the Nature Portfolio Reporting Summary linked to this article.

## Online content

Any methods, additional references, Nature Portfolio reporting summaries, source data, extended data, supplementary information, acknowledgements, peer review information; details of author contributions and competing interests; and statements of data and code availability are available at 10.1038/s41588-023-01637-y.

### Supplementary information

Supplementary Information Supplementary Note and Figs. 1–18.

Reporting Summary

## Data Availability

All datasets used for creation of the control repository could be obtained from the dbGAP or from the dedicated repository. The complete list of links is available in Supplementary Tables 1 and 2. The breast cancer cohort is available at dbGAP through phs000822.v1.p1. The TCGA cohort is available at dbGAP through phs000178.v11.p8. The SCoRe control repository could be accessed at http://dnascore.net. A tutorial and instructions on how to use the package and repository are provided at the ‘Tutorial’ tab of the SCoRe website.

## Code availability

Source code for SVDFunctions^30^ is available at https://github.com/alexloboda/SVDFunctions.
