# Supplementary material for: Public platform with 39,472 exome control samples enables association studies without genotype sharing
Source: Nat Genet. 2024 Jan 10;56(2):327–35. doi: 10.1038/s41588-023-01637-y (PMC10864173; doi:10.1038/s41588-023-01637-y)
Supplement: Supplementary file 1 — Supplementary Note and Figs. 1–18. [file 41588_2023_1637_MOESM1_ESM.pdf]

nature genetics

Technical Report

https://doi.org/10.1038/s41588-023-01637-y

Public platform with 39,472 exome control samples enables association studies without genotype sharing

In the format provided by the authors and unedited

Supplementary Note

1. Complimentary R-package 2
  1.1. Control selection algorithm 2
    1.1.1. Genotype matrix generation 2
    1.1.2. Genotype imputation 2
    1.1.3. Harmonizing the genotype matrices of case and control pool 5
    1.1.4. Subsampling control set using simulated annealing 5
  1.2. Framework and tools for control selection protocol 6
    1.2.1. Outlier detection and cohort PCA-normalization 6
    1.2.2. Multiple ancestry clusters 9
    1.2.3. Shareable data structure (YAML file) 12
    1.2.4. Control data access and control set genotype counts generation 12
    1.2.5. SCoRe server design 13
2. Datasets available through SCoRe 14
  2.1. Exome dataset details 14
  2.2. Exome sequencing data QC 18
3. Performance evaluation 18
  3.1. Method cross-validation 18
  3.2. Fine-scale ancestries matching in independent dataset 19
  3.3. Selecting controls for case cohorts with internal structure of subpopulations 21
  3.4. Cross-sequencing platforms control selection 23
  3.5. Selection of control samples from the specific sequencing platform 26
4. Case study. Breast cancer association study using SCoRe platform. 27
  4.1. Description of the dataset 27
  4.2. Rare variants with different thresholds 28
  4.3. Matching controls with shared genotypes 29
  4.4. Power calculations 30
5. Case Study. TCGA African-American cohort pan-cancer association study. 30
6. Case Study. Focal Segmental Glomerulosclerosis African-American cohort association study. 33
7. References 34

1. Complimentary R-package

SVDFunctions – a complimentary R-package consists of a collection of functions and utilities to be used on the local machine with case cohort for fast and reproducible preprocessing of the local genetic data for further control selection with SCoRe online platform. Specifically, we provide utilities to read VCF files, impute missing genotypes for performing SVD-based analyses and generation of the shareable non-individual level data in a form of a single YAML-file that can be uploaded to the dnascore.net for control selection.

1.1. Control selection algorithm

1.1.1. Genotype matrix generation

SVDFunctions implies local availability of case cohort genotypes in the form of VCF file. We provide the function *genotypeMatrixVCF()* that reads a raw or compressed (GZ or BGZ) VCF file and creates a matrix of genotypes for a prespecified set of variants. We provide two recommended sets of the LD-pruned common autosomal variants for each control exome dataset used in the study through the objects in the SVDFunctions package - *publicExomesDataset* and *finSwedDataset*.

Genotypes are encoded as 0, 1, 2 (HomRef, Het, HomAlt, respectively). Optionally, we provide control over quality filtering – DP and GQ metrics for genotypes, as well as allowed rate of missing values per variant.

The function *filterGmatrix()* is used to post-process the original genotype matrices and filter them using the sample and variant call rate filters as needed for a particular usage scenario.

1.1.2. Genotype imputation

Conventional approach to PCA in genetics replaces missing genotypes with mean genotypes for a given variant to ensure the completeness of the genotype matrix. In the presence of samples from multiple sequencing platforms in the same dataset, there are several distinct patterns of missing values that would suffer from systematic bias in case of applying the traditional solution for missing values imputation. Therefore, we developed a predictive model for imputing the missing genotypes that can learn such patterns from the given dataset itself. The imputation method does not use reference LD panels and is aimed to be a lightweight high-throughput replacement or in case when no good quality reference panel is available.

To eliminate missing genotypes, we use a modification of the Random Forest regressor. The process of imputation is completed at the time of reading a VCF file. For each variant in the file, genotypes of individuals with non-missing genotypes for this variant for the 100 nearest variants in the maximum window of 250kb, are used as sample features. The predictor for missing genotypes is built once for a variant and by default consists of 16 decision trees. Each tree is constructed after performing the sample bagging. At the time of training of the model, splits are performed on features with maximum information gain.

At the first step, a complete sample emerged as a result of bagging, consisting of pairs (sample, weight), enters the process of construction of a decision tree. The weight of each sample is set to 1.0 at this step. Then each feature and each splitting point is tested for being a maximum cut in terms of the information gain. Then samples are divided into two smaller bags depending on the selected split condition. In the case of missing value of the splitting feature for a specific sample, it appears in both bags with weights proportional to the frequency of each possible value calculated on the known values of the original bag. The procedure is repeated for each constructed node of the decision tree until either all samples reaching the node have the same value for the prediction feature, or there is no split available.

For each node we calculate the expected squared error in case of sampling from posterior multinomial distribution with Jeffrey's priors. Post pruning of the trees is performed using the errors. If such error in the node is smaller than the one expected if the split persists, then the left and right subtrees can be cut off.

The process of prediction starts from the root node of each decision tree. The descending in the tree is performed by following the conditions at each node. The solution is returned when the process reaches the leaf node. Weighted mean value of the samples at the node is returned. If the process of traversing reaches the node where the value of the splitting feature is not available for the predicting sample then the process splits into two. Returned values from the subtrees are mixed proportionally to the frequencies of the known values in the training sample that reached the node.

Therefore, we construct an entire genotype matrix with no missing values, keeping the information about which genotypes were imputed in a separate boolean matrix.

Unlike other solutions used to overcome the problem of the missing values in PCA/SVD, which use mean genotypes for a variant as a substitute for the missing genotypes, our approach delivers more accurate imputation quality that is critical for the downstream steps of case-control matching.

To evaluate the quality of the genotype imputation algorithm, we used 1000 Genomes OMNI array genotyping data for 2318, downloaded from ftp://ftp.1000genomes.ebi.ac.uk/vol1/ftp/release/20130502/supporting/hd_genotype_chip/ALL.chip.omni_broad_sanger_combined.20140818.snps.genotypes.vcf.gz . The imputation of missing genotypes at variants that could be used for control selection in SVDFunctions package (N=4,116 after intersecting with variants available in the 1000 genomes) was performed using *genotypeMatrixVCF()* . As a control experiment we used the conventional approach of replacing missing genotypes with a variant mean.

We performed 5-fold cross validation and estimated mean squared error. We divided all the samples from the dataset randomly into five equally sized buckets. Training the model on the four out of five buckets we tested the model on the remaining one. Each bucket has been used as a test cohort exactly one time implying five runs of the train-test procedure forcing each sample to be weighted equivalently in the resulting error. The setting with zero decision trees has been added as a control method. The control method predictor returns the mean value of the genotype per variant (**Supplementary Figure 1**).

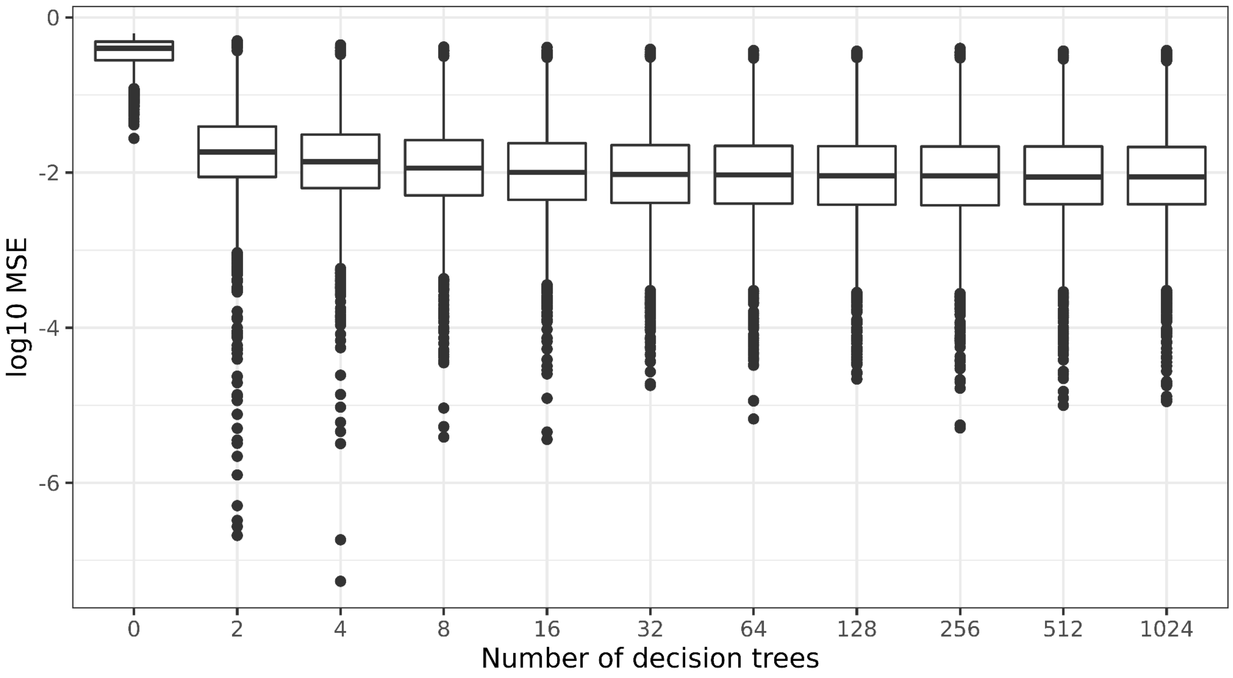

Supplementary Figure 1. Performance analysis of the SVDFunctions imputation tool. Log scaled mean squared error for the prediction of genotypes in 5-fold cross validation for the known good quality variants. First bar depicts the predictor with no decision trees and returning mean value, similar to the approach used in conventional PCA of genotype matrices. 16

decision trees is sufficient for producing good quality predictions in reasonable time, which is more practical when imputation is performed on a personal computer with few cores available. Central line represents median, upper and lower horizontal lines represent upper and lower quartile values.

The described above procedure of reading the VCF file is performed with *genotypeMatrixVCF()* function and imputation of the genotypes and creation of the genotype matrix are activated by default setting *impute=TRUE.*

As a result, the function produces a list of two matrices – a genotype matrix with imputed values and a boolean matrix indicating which of the genotypes were imputed. Additionally, the function returns summary details on the VCF reading and imputation process.

1.1.3. Harmonizing the genotype matrices of case and control pool

In case if not all variants from the control pool offered for ancestry matching are found in the case cohort, the following procedure is used to reconcile the case and control pool samples to the same basis. Original matrix U_{10} generated from the control pool is used to construct a matrix $\widehat{U_{10}}$ through the row elimination. The rows that should be eliminated correspond to the variants that are missing in the case cohort. Next, the coordinates of eigenvectors of case genotype vector projections in the basis $\widehat{U_{10}}$ should be obtained. This is achieved through inverting the matrix $\widehat{U_{10}}$. Throughout the manuscript we use U_{10}, implying that the same logic applies to $\widehat{U_{10}}$ if the basis harmonization procedure has been used. For better performance of the matching process and avoidance of possible computational errors due to close to singular matrix input after reduction, we require that at least 500 variants are available for the matching process in the local case cohort.

1.1.4. Subsampling control set using simulated annealing

Minimization of the BHEP statistic during the process of control sampling is achieved using a simulated annealing algorithm. A subset of a control cohort could be represented as a binary vector of the size of a complete control cohort with 0 indicating absence from the subset and 1 indicating presence in the subset. For a fixed subset size the number of 1s in the vector must be equal to the subset size. Neighboring solutions are selected in a way that two positions with different values in a vector are inverted keeping the number of ones and zeros the same.

The fitness of the control subset data points in the U_{10} to the case distribution is assessed with BHEP statistic. Simulated annealing is one of the conventional meta-heuristic algorithms for optimization of functions, including the ones with binary features (subset vector). As a result, the optimization algorithm returns a binary vector (subset) attempting to maximize BHEP statistic.

Simulated annealing can be interpreted as a hill climbing algorithm with a modification that enables preference of non-improving solutions with probability $p = e^{-\Delta E/T}$, where ΔE is the difference in optimizing function value and parameter T is referred to as "temperature", that decreases throughout the process. For our implementation we have selected the annealing schedule as follows: the temperature at the iteration $i + 1$ is defined by the equation $T_{i+1} = cT_i$. By definition, the value of the BHEP statistic is ranged between 0 and 1, therefore it is possible to set initial and final temperatures such, that the only parameter of the algorithm is left - a number of iterations N. We implemented this step by setting the Initial temperature T_0 to satisfy the condition of two subsets with maximal difference in BHEP statistics to be accepted with probability $\frac{1}{2}$. Maximal difference of BHEP statistics according to definition is 1, and given the probability of ½ we can estimate T_0 as: $T_0 = -\frac{\Delta E}{ln\, p} = \frac{1}{ln\, 2}$. On the other hand, final temperature T_N is chosen so that difference in function values of size of machine precision (ε) would be accepted with probability $\frac{1}{2}$, meaning $T_n = -\frac{\Delta E}{ln\, p} = \frac{\varepsilon}{ln\, 2}$. Parameter c is then calculated using T_0, T_N and number of steps N, leaving N as a single parameter of the algorithm.

Now, using this parameter, we can establish a complete process of optimization of the target value which is the BHEP statistic for a fixed subset size. For a fixed control cohort, the number of iterations is the only parameter that determines the running time of the algorithm. In our R-package function *selectControls* the parameter is called 'iterations' and by default is equal to 10^5.

1.2. Framework and tools for control selection protocol

1.2.1. Outlier detection and cohort PCA-normalization

As in any association study involving shared genotypes, outliers should be identified and removed from the analysis and the case cohort entering the analysis should represent relatively homogeneous ancestry. Although these steps could be performed manually before using our

algorithm, we developed a data preprocessing protocol that performs these steps in a situation where control genotypes are not available.

First, on the local machine, PCA is performed only on the cohort of cases. To remove outliers, we first run the k-nearest neighbors anomaly detection algorithm. The number of samples to be removed could be predefined based on visual peculiarities of principal components picked up by a user on a local machine. Then, we use the approach similar to the subsampling process implemented during control set selection to get the distribution of case data points closer to Gaussian. We fix both μ_P and K (**Methods, Shareable Data Generation**) and run simulated annealing to select a subset of the size of 95% of the original case cohort with minimized BHEP statistic. This step is optional and could be used to increase the size of the selected control cohort at the cost of decreasing the size of the case cohort (**Supplementary Figure 2**).

We illustrate the outlier removal protocol with a cohort of 244 breast cancer samples of European descent (dbGAP accession id: phs000822.v1.p1).

The cohort includes individuals with early onset (<35 y.o.) breast cancer. Patient went through the preliminary screening of previously known risk germline variants in *BRCA1* and *BRCA2*. All individuals were sequenced using Agilent exome capture

First, a VCF file with 251 samples was converted to a genotype matrix containing genotypes for variants from the list provided by the package SVDFunctions and missing genotypes imputed using Random Forest (**Genotype Imputation**). Then, the variants and samples in the genotype matrix were subjected to quality filtration using *filterGmatrix* function with minimum variant call rate set to 0.95 and minimum sample call rate set to 0.9. Then PCA matrix was constructed using *gmatrixPCA()* function from the R package with the control pool basis obtained from the SVDFunctions object *publicExomesDataset*.

Second, outliers were removed internally by calling the function *prepareInstance* that is used to generate shareable data that later could be uploaded to the SCoRe server. Each cluster is processed with a two-step outlier elimination algorithm. First, outliers are detected using K-nearest neighbors (KNN) anomaly detection algorithm. Next, we estimate the parameters of multivariate normal distribution for cases by running maximum-likelihood estimator (MLE) on the case coordinates, producing mean and covariance. Next, the multivariate normal distribution filter is applied, which eliminates outliers based on maximization of the BHEP statistics. BHEP statistics assesses similarity between observed case distribution and the fitted multivariate normal distribution. The maximization process is implemented through the simulated annealing algorithm, similarly to the control selection process (**Subsampling control set using simulated**

annealing). A user can provide the share of samples to be eliminated by each outlier detection algorithm by passing values to parameters *knn_drop* and *normalize_drop.*

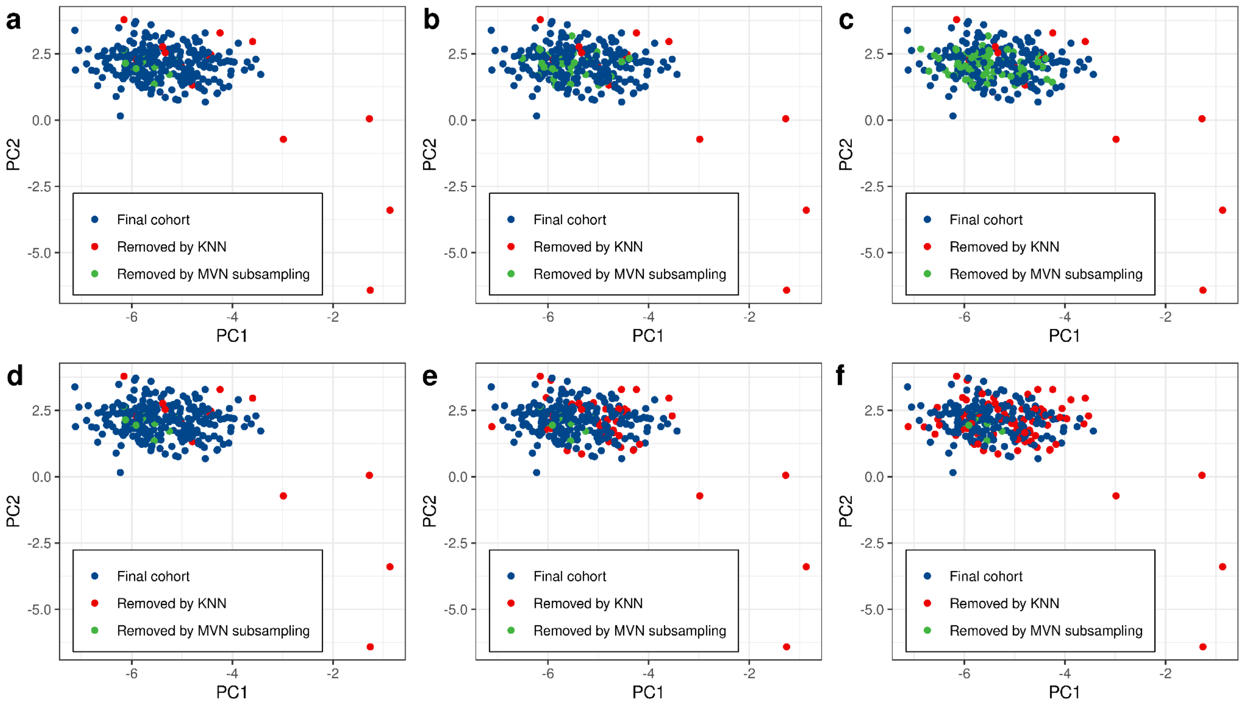

Supplementary Figure 2. Outlier elimination algorithm applied with different thresholds. (a) - (c) KNN removal is fixed at the rate 0.05, while MVN subsampling is set to 0.05, 0.15, 0.3 correspondingly. (d) - (f) –- MVN subsampling rate is set to 0.05, while KNN dropout rate is set to 0.05, 0.15, 0.3 correspondingly.

1.2.2. Multiple ancestry clusters

The single Gaussian model can be easily extended to the Gaussian mixture model, supporting the presence of several population clusters. To keep for the downstream association studies as large case clusters as possible, we use hierarchical Gaussian clustering. It represents clustering of the case cohort as a binary tree, where each node, including internal ones, represents a parametrized multivariate normal distribution. The control selection process is then performed for each node independently. If it is possible to select controls for the larger case cluster, represented as a parental node, then both subtrees could be excluded from the return results. We set a specific set of rules of multi-cluster selection of controls to form the returnable results to ensure that no overlapping sample will be used for the association study and the selected control dataset has optimal quality

In the presence of multiple ancestry clusters in the case cohort, the MClust package is used to perform hierarchical clustering on coordinates of case samples in the basis U_{10} (**Supplementary Figure 3a**).

Each node in the clustering object, including internal ones, corresponds to a subset of case samples. At each node the outlier elimination step is performed independently (**Outlier detection and cohort PCA-normalization**). Then, shareable data is created for every cluster and could be sent to the remote control pool. On the remote server, for each case subset the control selection process returns an appropriate subset of control samples. Note, the same control sample can be present in more than one of such subsets (**Supplementary Figure 3b**).

We developed a procedure ensuring that the returned control subset, when used for association testing on the local machine, participates in the analysis only with non-overlapping sets of samples. First, control subsets could not be returned along with control subsets for cluster-descendants (i.e. control set for case cluster 1U2 could not be returned along with control set for clusters 1 or 2). Second, we provide information on genotype counts for all permitted for return clusters and for sample overlaps between them (i.e. in case if control sets are returned for case clusters 1U2 and 3, genotype count file will include the data on each of these subsets and the overlap between control set for case cluster 1U2 and case cluster 3).

From each subtree the pairs of non-overlapping case cohorts and corresponding matched control sets forming association tests with inflation factor below soft threshold with maximum number of used samples from control pool is returned. It can be set by using parameter mergeCoef of the function selectControlsHier that it is favorable to consider merged case clusters as case cohort even if the matched control set is smaller than union of control sets for individual clusters by constant factor. If some of the case clusters do not participate in one of such pairs then control set below hard threshold is returned for this cluster. If two pairs contain the same case cluster then the one with minimal inflation factor is returned (**Supplementary Figure 3c**).

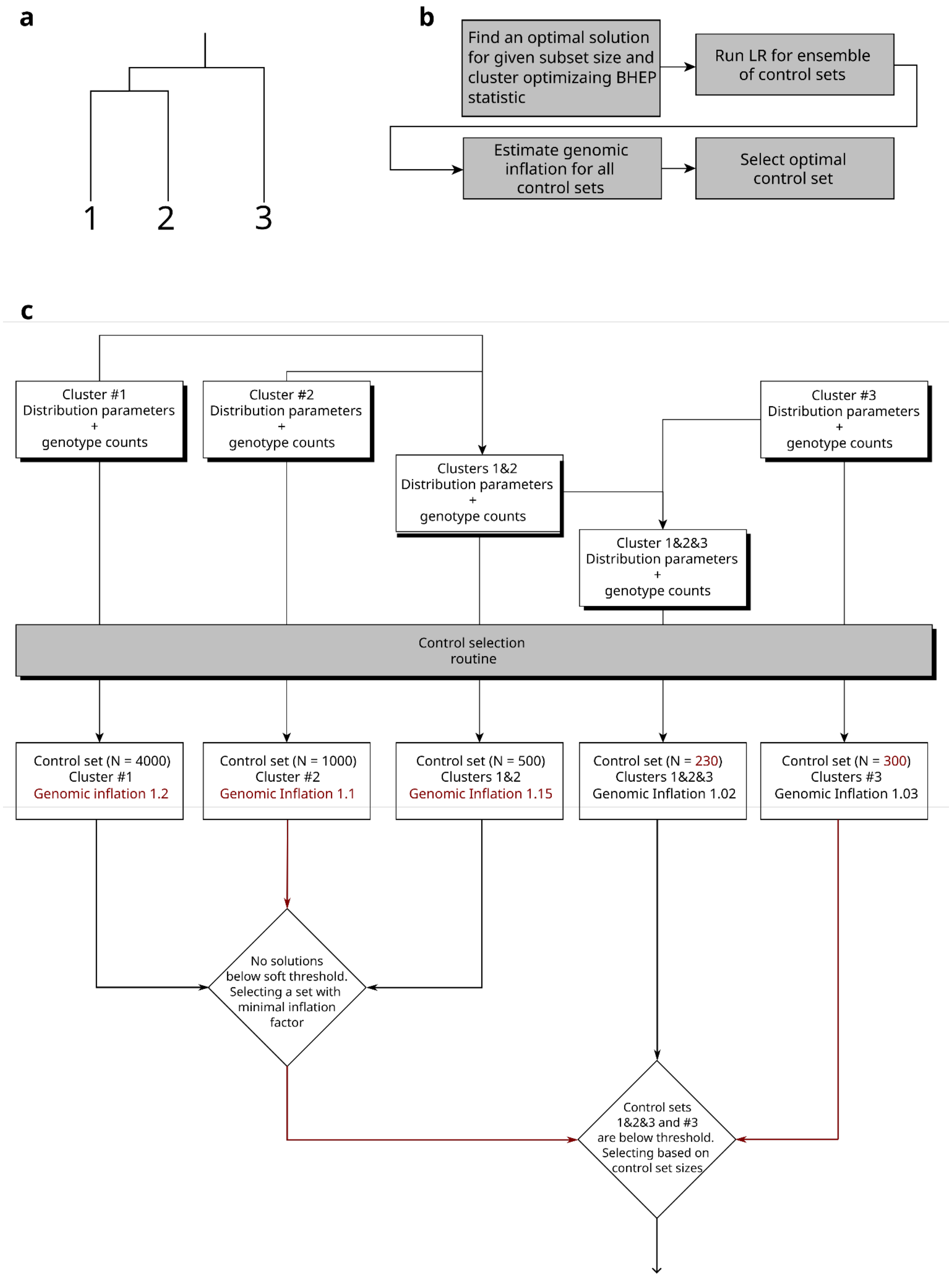

Supplementary Figure 3. Selecting controls in multiple ancestry clusters setting. Figure (a) describes the hierarchical structure of given three clusters, figure (b) depicts the procedure at which the best control set is selected. Finally, figure (c) shows what data are precomputed for each cluster and for the merged clusters and for what data the controls selection routine is run.

1.2.3. Shareable data structure (YAML file)

SVDFunctions package contains tools for building a YAML file that is used as an effective structured framework for transmission of shareable data needed for control selection to a remote server. First, it contains a subset of variants found in cases from the list of variants available for matching supplied through *SVDFunctions*. Second, the population structure of the case cohort is stored as a tree. And, finally, each node cluster, including internal ones has a separate record in the file, containing genotype counts of each variant and cluster distribution parameters under the assumption of multivariate normality of sample coordinates in control pool basis within clusters.

Importantly, our algorithm assumes that a case cohort should be represented by a relatively homogenous ancestral cluster, which is a common scenario in genotype sharing association studies as well. Therefore, if multiple ancestries are present in the case cohort it is important to separate them and generate shareable data for each cluster. We implemented a hierarchical clustering function *estimateCaseClusters()* that detects clusters in case cohort PCA space. It uses MClust library with hierarchical Gaussian mixture model based on Bayesian information criterion (BIC) to detect an optimal number of clusters.

For each case cluster SVD is performed on a genotype matrix with imputed values. Additionally, summary genotype counts are estimated from the genotype matrix with missing values, to ensure that imputation does not introduce bias into the allele frequencies.

The process of generation of shareable data for the entire case cohort was automated with function *prepareInstance()*. It takes as an input both genotype matrices with and without imputed genotypes for the case cohort and clustering object returned by *estimateCaseClusters()*. Eigenvectors of the matrix constructed from the projected vectors of genotype matrix into U10 basis corresponding to a cluster of cases and summary genotype counts for each cluster are formatted into a YAML file in concordance with cluster hierarchy, so that it could be recovered on the control server. Further, the generated YAML file could be directly uploaded to dnascore.net as input for control selection.

1.2.4. Control data access and control set genotype counts generation

VCF files with thousands of samples could not be efficiently accessed for summary allele frequency calculations in a high-throughput manner. Therefore, we developed a binary format for fast query of genotype counts. VCF files for Public Exomes and Nordic

dataset control samples were converted into binary databases with *scanVCF()* function, allowing genotype and variant quality filters. Such binary database could be efficiently accessed from the cloud environment by multiple workers to maintain queries from several users at the same time with no notable slow down.

Selected control set (**Supplementary Figure 3**) is used to query binary database with *scanBinaryFile()* function to obtain summary genotype counts using user-provided genotype quality filters. When using control selection for multi-cluster input it is possible that control sets for some clusters will overlap (i.e. some control samples will fit in more than one cluster control set). Therefore, in case of selecting 3 control sets as optimal (**Supplementary Figure 3**, Control sets for cluster #1, cluster #2, cluster #3), for output from the control
database will contain information about summary genotype counts for each independent cluster and all possible overlaps: #1∩#2, #1∩#3, #2∩#3, #1∩#2∩#3.

Information about overlapping samples could be used in two ways. First, counts for overlapping samples could be simply subtracted from counts for individual clusters control sets. In this way independent, non-overlapping control sets will be obtained. Second, it is feasible to use association test strategies for overlapping control samples[4,5].

1.2.5. SCoRe server design

SVD-based Control Repository (SCoRe) is implemented using R Shiny package[6] and could be accessed at http://dnascore.net . SCoRe is hosted at Google Cloud environment providing users with access to two control datasets described in the main text – Global Populations and Nordic exomes.

To use the platform, user needs to install R-package "SVDFunctions" from https://github.com/alexloboda/SVDFunctions and create a genotype matrix for the set of variants provided for matching for each of the two datasets, starting from the case VCF file (*genotypeMatrixVCF()* function), perform genotype matrix quality filtration to eliminate poorly covered samples or variants (*filterGmatrix()* function) and prepare the YAML file with shareable data (**Shareable data structure (YAML file)**) . Resulting YAML file could be directly uploaded to http://dnascore.net .

SCoRe will select the control set, generate the QQ plots for each cluster (and cluster compositions), and report genomic inflation estimated on the variants that were used for matching.

If a file with either a list of variants or gene names for which information from the control set should be returned is provided – SCoRe will also provide a link to download control genotype count data.

Each session has a unique ID and the user will be notified over the e-mail about the status of the job. Session could be revisited using the link from the e-mail.

Additional security precautions were implemented to exclude possibility of individual genotype disclosure, by finding two control sets, different by only one sample. We set a hard threshold on a minimal number of selected controls (100 samples) to deliver allele frequencies to the user. Also, we clustered controls in the PCA space into clusters of 16 samples(using ELKI[1] same-size k-means clustering algorithm) and fixed them, so that the minimal difference between any two control sets would be 1 indivisible cluster (16 fixed samples), so the individual level genotypes could not be uncovered in any way.

DP, GQ parameters for controls genotype quality could be specified to match case dataset QC standards. We provide users with an option to specify desired matching accuracy (λ, genomic inflation).

Queries to the database could be asking for variant based frequencies or cumulative counts of rare variants per gene. For variant based queries we provide summary allele counts for any variant except singletons in chosen control set. Instead, singletons could be obtained in the form of summary per gene. To ensure there is no bias in inclusion of variants into the rare variant association tests, we provide an option to specify MAF threshold using publicly released allele frequencies from gnomAD, in this way, MAF is not biased by number of samples in case or control set.

Illustrated tutorial and test data are available within the “Tutorial” section at http://dnascore.net .

2. Datasets available through SCoRe

2.1. Exome dataset details

Supplementary Table 1. Samples available for control matching through SCoRe platform Global Populations dataset

Project	Sample Count	Access	Majorly Represented Ancestries	Ascertainment	Sequencing Depth
**1000 Genomes**	1544	https://www.internationalgenome.org/data/	AFR, AMR, EAS, EUR, SAS	-	37.5X
**Myocardial Infarction Genetics Exome Sequencing Consortium: Italian Atherosclerosis Thrombosis and Vascular Biology**	3438	phs000814.v1.p1 (dbGAP)	EUR	healthy subjects without a history of thromboembolic disease. They were enrolled from among the blood donors or staff of the participating hospitals.	32X
**Autism_Daly_NIMH/NHGRI(ARRA) WholeExome**	216	-	EUR	No ASD	45.5X
**NHLBI Exome Sequencing Project**	4387	http://evs.gs.washington.edu/EVS/	EUR, AFR	https://www.ncbi.nlm.nih.gov/projects/gap/cgi-bin/study.cgi?study_id=phs000400.v3.p1	34.3X
**NHGRI_Autism_Daly**	151	-	EUR	No ASD	45X

**Myocardial Infarction Genetics Exome Sequencing Consortium: Ottawa Heart Study**	999	phs000806.v1.p1 (dbGAP)	EUR	Asymptomatic for cardiovascular disease elderly persons without cardiovascular disease history were selected as controls (>=65 years of age for males, >=70 years of age for females) and recruited by an advertising campaign in the Ottawa community.	32.9X
**T2Genes study**	5797	phs001552.v1.p1 (dbGAP)	AFR, AMR, EAS, EUR, SAS	Controls were participants who had no prior history of type-2 diabetes, had normal glucose levels or HbA1c levels < 6.0%	32.6X
**Total**	16532				

Supplementary Table 2. Samples available for control matching through SCoRe platform Finnish and Swedish exomes dataset.

Project	Sample Count	Access	Ancestries	Ascertainment	Mean Sequencing Depth
**1000 Genomes**	49	https://www.internationalgenome.org/data/	FIN	-	40.1X
**AD FINRISK**	204	https://www.ncbi.nlm.nih.gov/pubmed/29165699	FIN	No Alzheimer's disease	38.4X
**AD Twins Sistonen**	325	https://thl.fi/en/web/thl-biobank/for-researchers/sample-collections	FIN	No Alzheimer's disease	36.7X
**Controls Holtman**	6037	https://thl.fi/en/web/thl-biobank/for-researchers/sample-collections	SWE	-	38.1X
**Controls NFBC**	745	https://thl.fi/en/web/thl-biobank/for-researchers/sample-collections	FIN	-	54.6X
**FINRISK**	8994	https://www.ncbi.nlm.nih.gov/pubmed/29165699	FIN	-	35X
**Health 2000**	1673	https://thl.fi/en/web/thl-biobank/for-researchers/sample-collections	FIN	-	52.8X
**IBD Finrisk (controls)**	662	http://www.type2diabetesgenetics.org/projects/t2dGenes	FIN	No IBD	35.1X
**T2D Fusion**	806	http://www.type2diabetesgenetics.org/projects/t2dGenes	FIN	No T2D	41.7X
**T2D**	921	http://www.type2diabetesgenetics.org/projects/t2dGenes	FIN	No T2D	34X
**T2D Metsim**	952	http://www.type2diabetesgenetics.org/projects/t2dGenes	FIN	No T2D	35.3X
**UK10K**	1572	https://www.uk10k.org/data_access.html	FIN	-	38.2X
**Total**	22940				38.3X

2.2. Exome sequencing data QC

Both Global Populations and Finnish&Swedish exome datasets were subjected to per sample and per variant quality filtration with further construction of genotype matrix for common autosomal LD-pruned variants. Genotype matrices were further used for SVD and case-control matching experiments. SVDFunctions R-package provides all functionality needed for running this workflow.

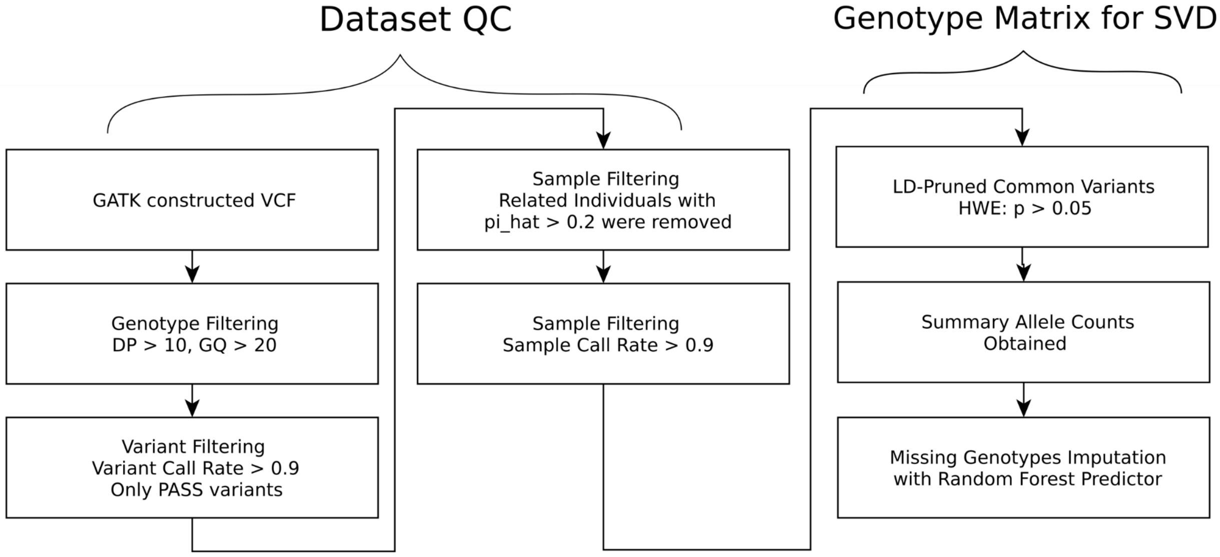

Supplementary Figure 4. Exome sequencing data QC.

3. Performance evaluation

3.1. Method cross-validation

We evaluated the performance of the algorithm in selecting the controls from the appropriate ancestry through the series of cross-validation experiments. First, using Public Exomes dataset, random 500 samples from each continental ancestry were set aside as a case cohort with the rest becoming a pool of controls. The control selection algorithm was then applied to the simulated cohort of cases and resulting control sets were evaluated for proportion of samples from the same continental ancestry (true positive) and other ancestries (false positives). In this experiment, soft threshold genomic inflation was varied from 1 to 10 with 1 corresponding

to the targeted “ideal” case-control matching and 10 corresponding to selection of the entire control pool as a matched control dataset (**Supplementary Figure 5a**).

Additionally, parameter sensitivity for the size of the case dataset was tested (**Supplementary Figures 5b, 5c**).

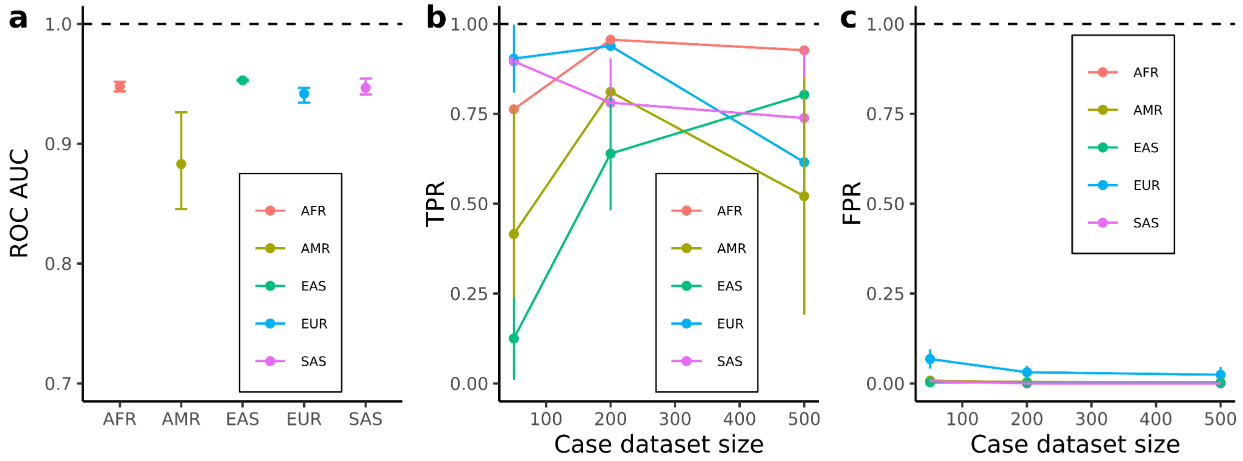

Supplementary Figure 5. ROC analysis for ancestry selection and sensitivity to the size of a case cohort.

(A) Area under the ROC curve. True positive – selection of the control sample from the same continental ancestry as case cohort. Estimates are based on 10 random case cohort selections for each value of genomic inflation – 1, 1.02, 1.05, 1.1, 1.2, 1.3, 1.5, 1.7, 2, 5, 50; (B) True positive rate is generally increasing or remaining the same as the case cohort size increases; (C) False positive rate is low, ensuring no samples of other than target ancestry are not selected for the control set. TPR and FPR calculated based on target genomic inflation of 1.05 in 10 random case groups. Points in the figure represent mean of 10 simulations and whiskers represent standard deviation.

3.2. Fine-scale ancestries matching in independent dataset

Next, we evaluated the ability of our approach to select controls for fine-scale ancestries and discriminate the situations when no controls from the specific fine-scale ancestry are available in the control pool.

As a case cohort we used 1708 unrelated samples from the 1000 Genomes, genotyped using the OMNI microarray. Such a scenario represents both the fine-scale ancestry control selection challenge and the selection of controls for the datasets that were not jointly processed, and even were generated using two different genotype discovery techniques.

Supplementary Figure 6 represents results of the control selection for each fine-scale ancestry from 1000 genomes. Bottom panel highlights the cohorts for which the selection of controls is impossible within the defined hard threshold ($\lambda \leq 1.05$).

We confirmed that such a result is consistent with absence of the control candidates in Public Exomes dataset from the local subpopulations that would not be returning the results using our algorithm. Conventional PCA shows no control candidates aligning the case cohort genotypes (**Supplementary Figure 6**).

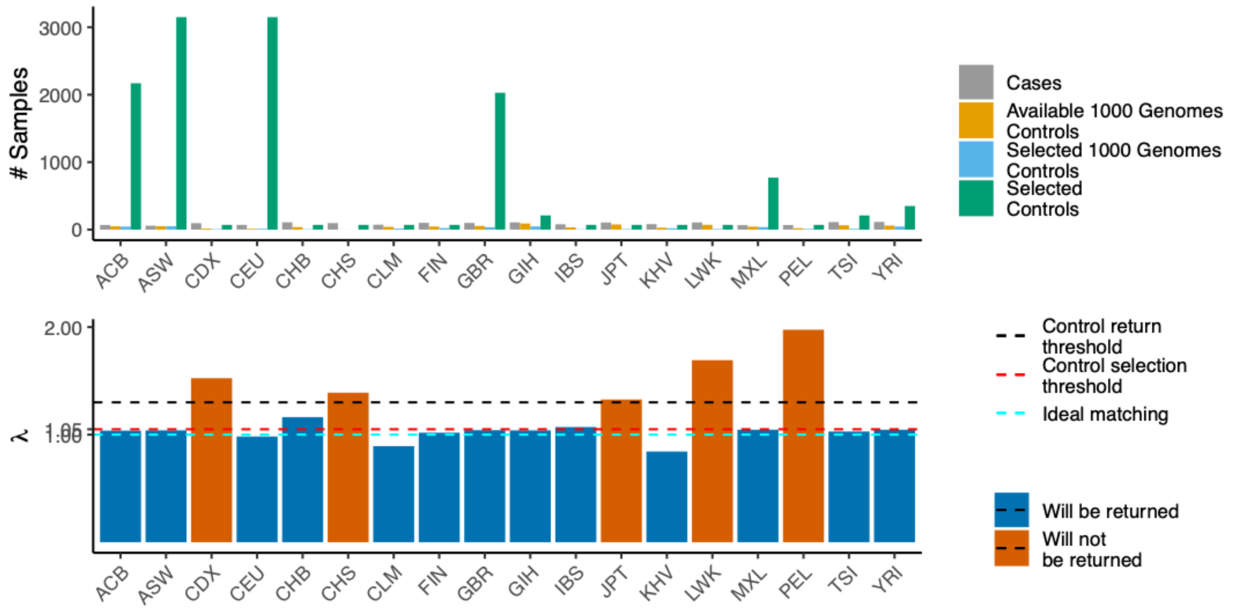

Supplementary Figure 6. Fine-scale ancestry matching using 1000 Genomes microarray-based data as dummy case dataset. Top: Summary of control selection exercise for fine-scale ancestries present in 1000 Genomes data. Some 1000 Genomes samples are also present in the control pool (Public Exomes dataset). Bottom: Control candidates for some of the fine-scale ancestries are not available in the control pool resulting in inability to select matched control delivering

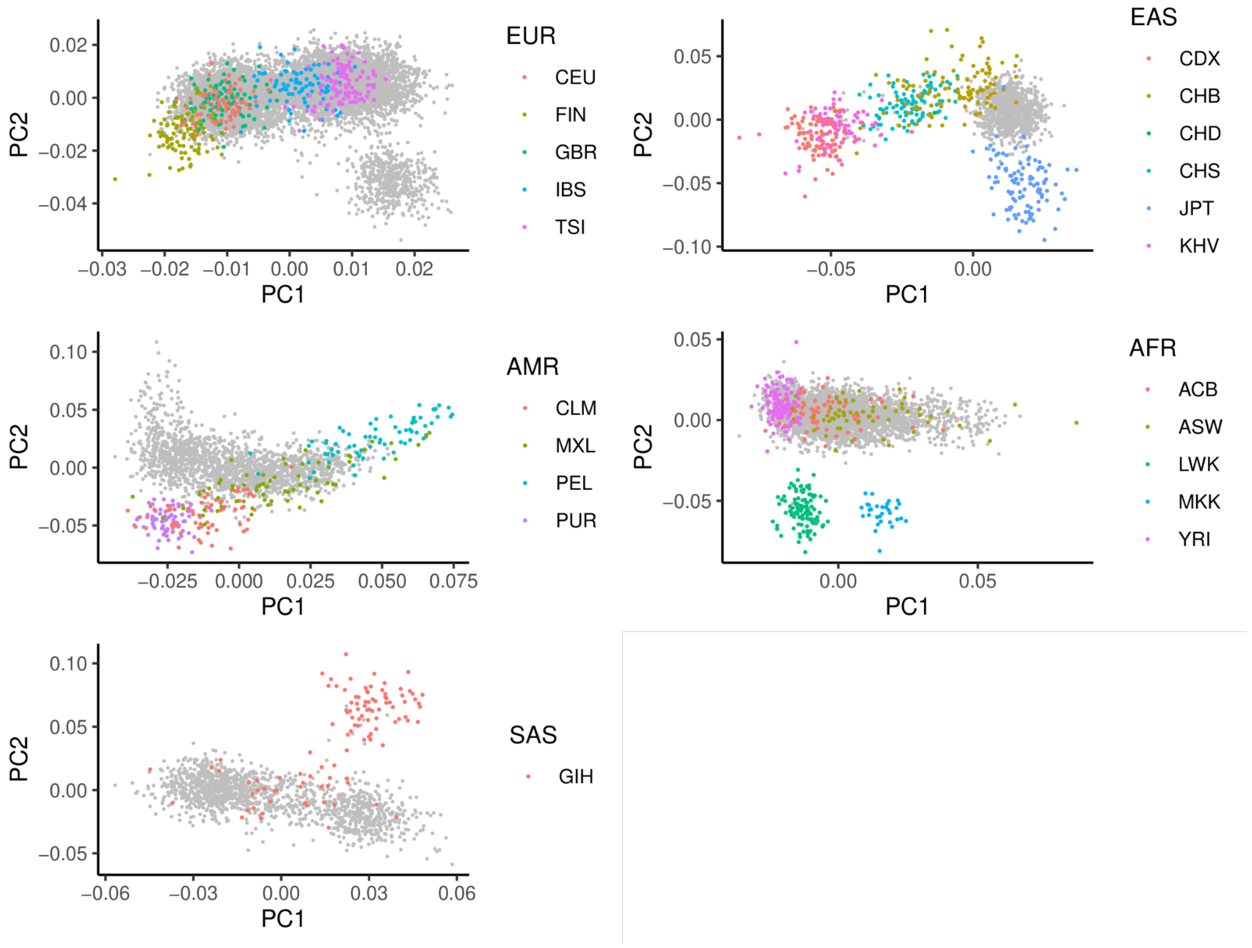

Supplementary Figure 7. Joint PCA of the simulated case cohorts from each subpopulation from 1000 Genomes with a pool of controls.

3.3. Selecting controls for case cohorts with internal structure of subpopulations

Next, we illustrate the selection of controls for a cohort of European population, containing samples from multiple local subpopulations and different genotype discovery technique from the control pool. We used the 1000 genomes European cohort for this experiment. First, the VCF file for 1000 Genomes OMNI array genotyping data was downloaded from (ftp://ftp.1000genomes.ebi.ac.uk/vol1/ftp/release/20130502/supporting/hd_genotype_chip/). Related individuals with PI_HAT>0.2 were excluded from the dataset (only one sample of the related cluster was kept). The resulting dataset with 459 European samples was converted to genotype matrix. Clusters within the case dataset were identified using *estimateCaseClusters* function and outliers were removed by running *prepareInstance* with default parameters. Public Exomes dataset was used as a pool of controls. Results of control selection for the multi-cluster input are presented in **Supplementary Figure 8**.

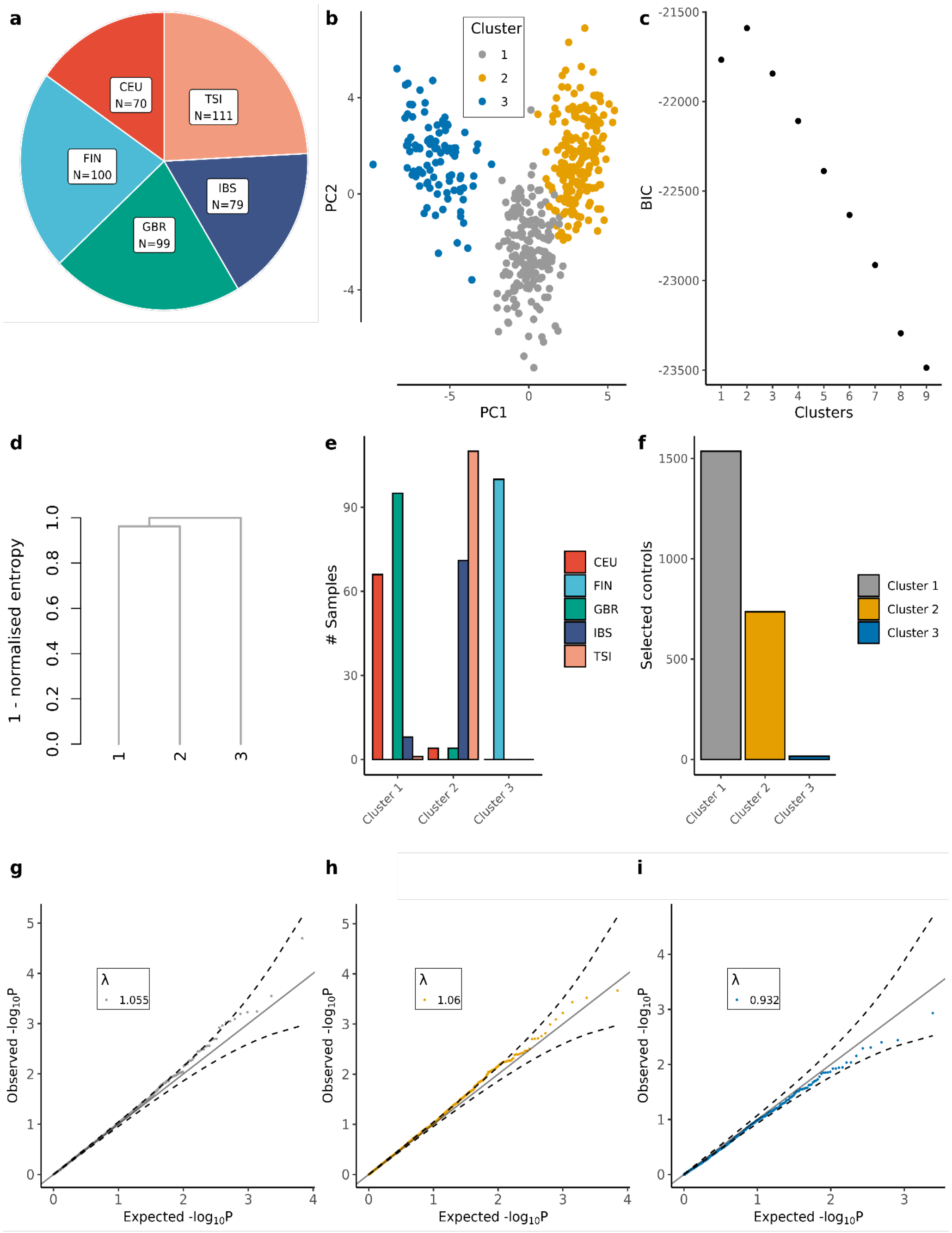

Supplementary Figure 8. 1000 Genomes European case cohort clustering and hierarchical control selection. (a) Composition of fine-scale ancestries in European continental ancestry cohort of 1000 Genomes data; (b) PCA in the space of case cohort and clustering highlights with clustering performed in basis of control PCA space; (c) Bayesian information criterion used in underlying Mclust clustering algorithm; (d) Case cluster hierarchy; (e) Cluster composition with respect to local European ancestries; (f) Control selection results for each cluster. Cluster 2, composed primarily of Finnish-descent samples has only few adequately selected controls as they are almost absent in the control pool; (g-i) QQ-plots for association tests with the selected control cohorts for each fine-scale ancestry cluster.

3.4. Cross-sequencing platforms control selection

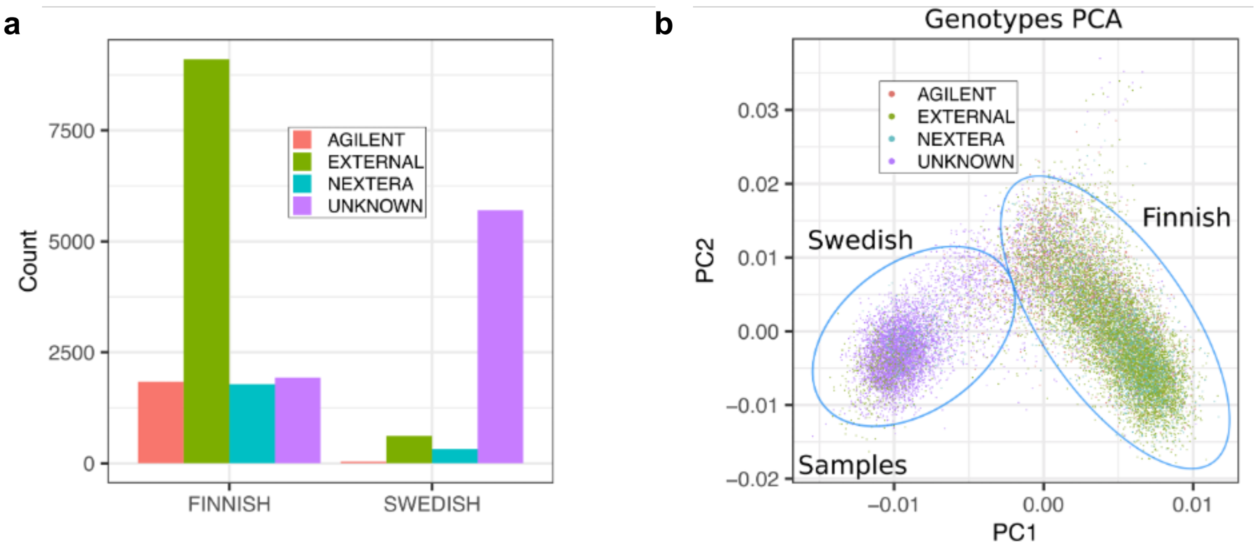

Supplementary Figure 9. Sequencing platform complexity. (A) Sequencing platform composition and (B) Conventional, genotype-based PCA of Finnish and Swedish exome dataset (N=22,940).

We performed a test showing capability of the method to produce high-quality results for usage scenarios when case and control cohorts were sequenced using different exome captures. First, as a case cohort was constructed from all NEXTERA samples from the Nordic dataset were selected (**Supplementary Figure 10a-b**). Genotypes then were filtered using 0.9 as variant and sample call rate thresholds. Then the U_{10} basis was constructed based on the genotypes of the remaining dataset which was used as a pool of controls.

After default filtering routines performed 2454 case samples were left and proceeded to the control matching step (**Supplementary Figure 9c**). With no clustering within the case cohort control set of 1507 samples were matched. Among these samples only 62 are reported as Swedish (**Supplementary Figure 9d**). Resulting association study (**Supplementary Figure 9e**) shows desirable level of inflation of QQ plot showing the evidence of importance of quality control protocols.

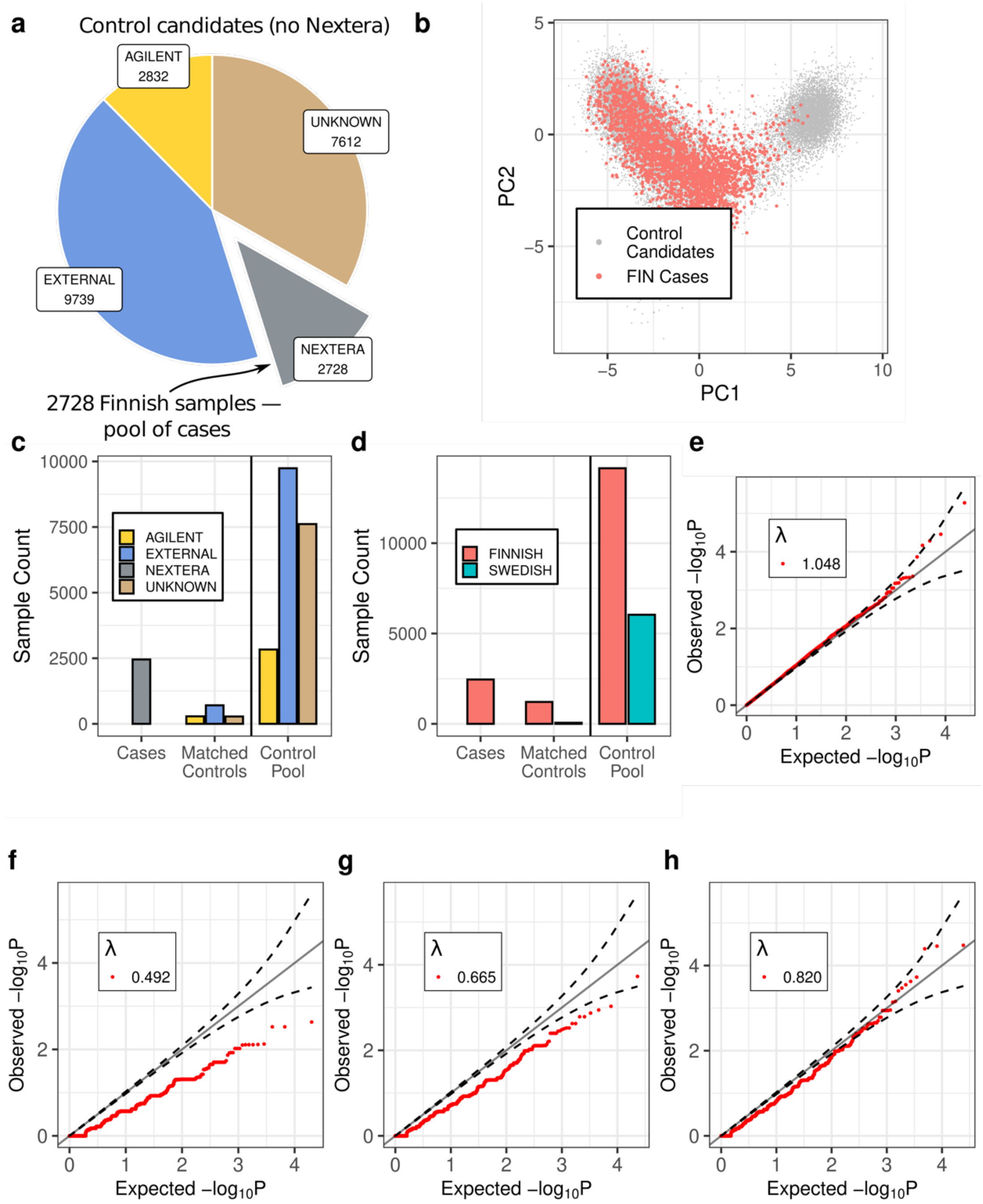

Supplementary Figure 10. Multi-platform association study using SCORE platform. (a) Platform composition of the Nordic dataset. (b) Principal component analysis for case and control cohorts in U10 basis of controls. (c) Number of matched controls compared to the number of cases and control pool across all sequencing captures. (d) Populational composition of case cohort, matched controls and control pool based on self-reported populational affiliation. (e) Association study conducted on the variants used for matching. (f-h) Gene-based rare synonymous variants association study (minor allele count in controls = 1, <=2, <=3). In panels (e-h) solid line represents a diagonal, dashed lines indicate 95% confidence interval, two-sided Fisher's exact test was used.

To perform this case study, we have selected the Finnish samples from 1000 Genomes that are a part of public exomes dataset (**Supplementary Figure 11a**). All the samples were sequenced using AGILENT exome capture. Then we have selected the Nordic dataset and removed all AGILENT samples from the control pool. *PrepareInstance* function from our R-package was used to perform variant quality control, no samples were removed by outlier detection algorithm due to small sample size of the case cohort. Then the control set for the case cohort was matched (**Supplementary Figure 11b-d**). All sequencing platforms from the Nordic dataset are present as a part of the control set (**Supplementary Figure 11e**).

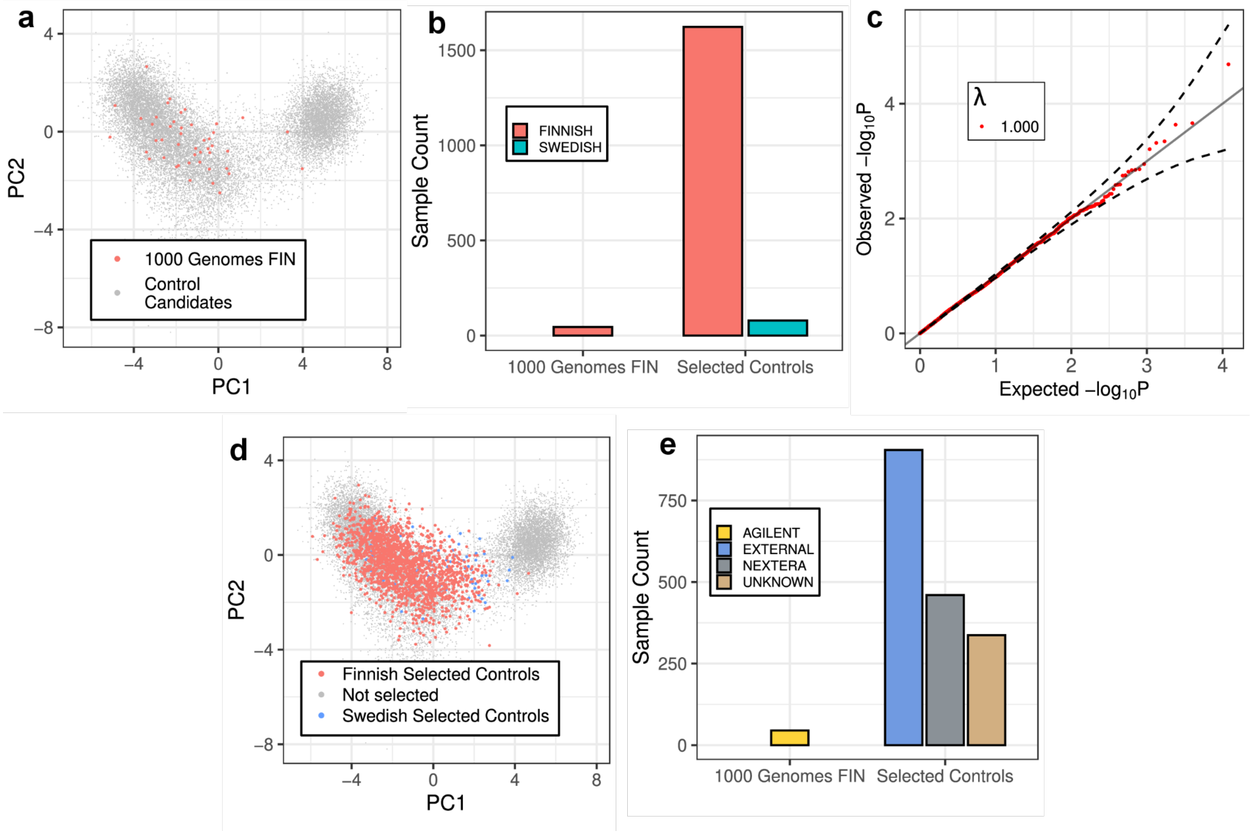

Supplementary Figure 11. Case study: cross-platform association study. (a) Principal component analysis performed on both case and controls cohorts showing projections of the samples in the U10 matrix constructed for the nordic dataset. (b) Populational stratification in both cohorts based on self-reported metadata. (c) QQ-plot of test statistics of association studies performed on synonymous variants that were not used in matching process - solid line represents a diagonal, dashed lines indicate 95% confidence interval, two-sided Fisher's exact test. Raw, unadjusted p-values reported; (d) Population stratification of selected controls showing self-reported population affiliation of samples. (e) Sample counts of each sequencing capture to be found in case cohort and matched controls.

3.5. Selection of control samples from the specific sequencing platform

We provide the tools for simple construction of the call rate matrices from the VCF files and the file with sequencing intervals. The function *callRateMatrixVCF()* builds a matrix of call rates per sample per region, given a set of genomic regions. For each region provided, all the variants within the range are scanned and the missing genotype rate is computed for each sample. The resulting matrix consists of rows corresponding to regions and columns – to samples. An entry value represents the call rate for a sample in a particular genomic region, given the DP and GQ filter thresholds. Usually, the regions could be taken from the exome capture BED-file.

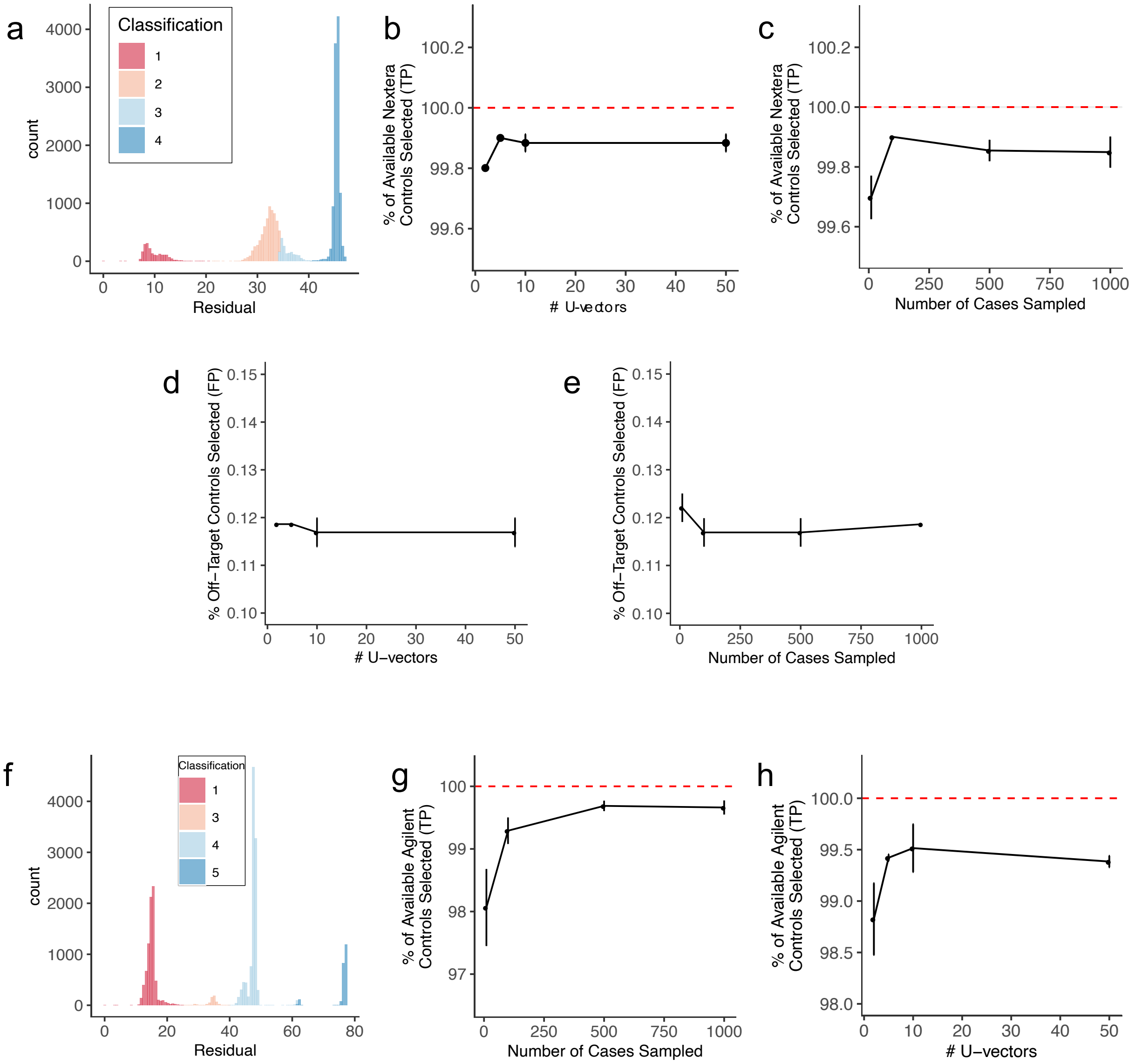

Supplementary Figure 12.Gaussian model fitting and parameter sensitivity for platform selection workflow. (A) Distribution of residual vector norms in Nextera “cases” experiment, colored with respect to Gaussian clusters identified by Mclust algorithm. (B-D) True positive and

false positive proportions in 100 simulation runs for selecting Nextera control candidates. (F) Distribution of residual vector norms in Agilent “cases” experiment, colored with respect to Gaussian clusters identified by Mclust algorithm. (G-H) True positive proportions in 100 simulation runs for selecting Agilent control candidates. In the figure, points represent mean of 100 simulations and whiskers represent standard deviation.

4. Case study. Breast cancer association study using SCoRe platform.

4.1. Description of the dataset

Test dataset of individuals with breast cancer was obtained from dbGAP (access ID: phs000822.v1.p1). Original cohort included individuals with early onset breast cancer (<35 years), pre-screened for known risk variants in breast cancer susceptibility genes. The cohort included individuals of European ancestry. Agilent v6 exome capture was used with mean coverage across the cohort 38X.

4.2. Rare variants with different thresholds

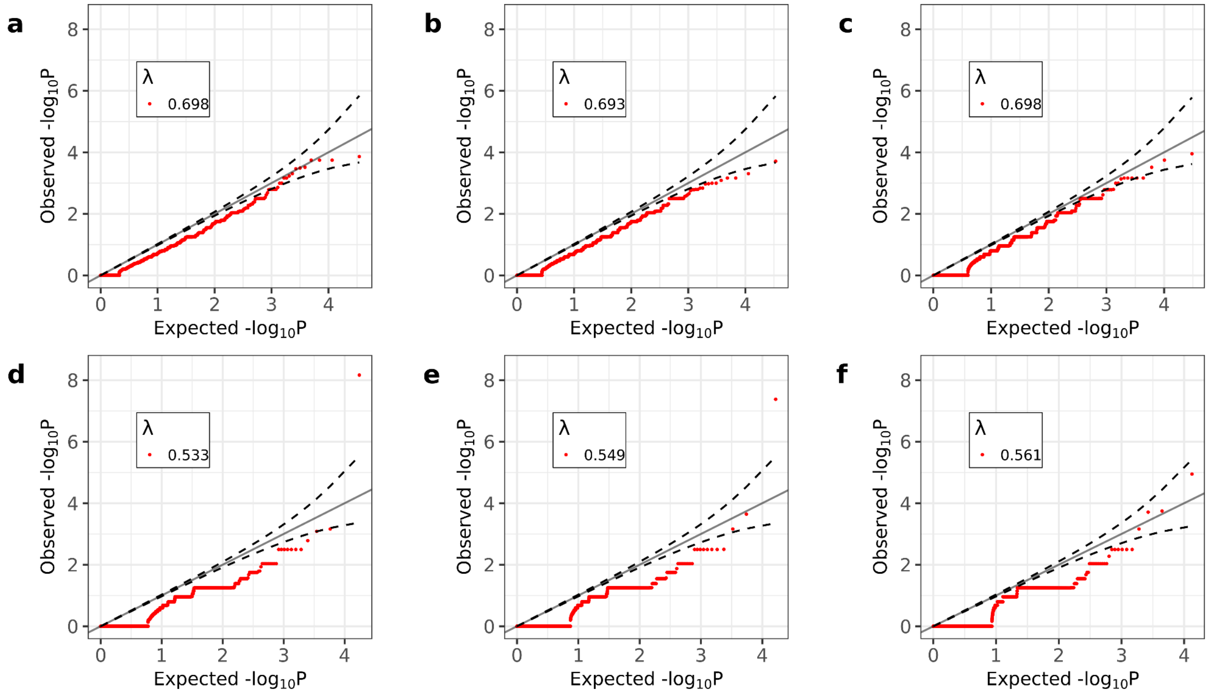

Supplementary Figure 13. Rare variant analysis for BRCA dataset with different minor allele frequency thresholds: (a) 10^{-3}, (b) – 10^{-4}, (c) – 10^{-5}. Solid line represents a diagonal, dashed lines indicate 95% confidence interval. Two-sided Fisher's exact test was used. Raw, unadjusted p-values reported

4.3. Matching controls with shared genotypes

Shared-genotypes PCA was performed on a joint dataset of breast cancer cohort and Public Exomes dataset. Controls were matched through subsampling of the control pool to match the observed distributions of PC1-PC4 (**Supplementary Figure 14**).

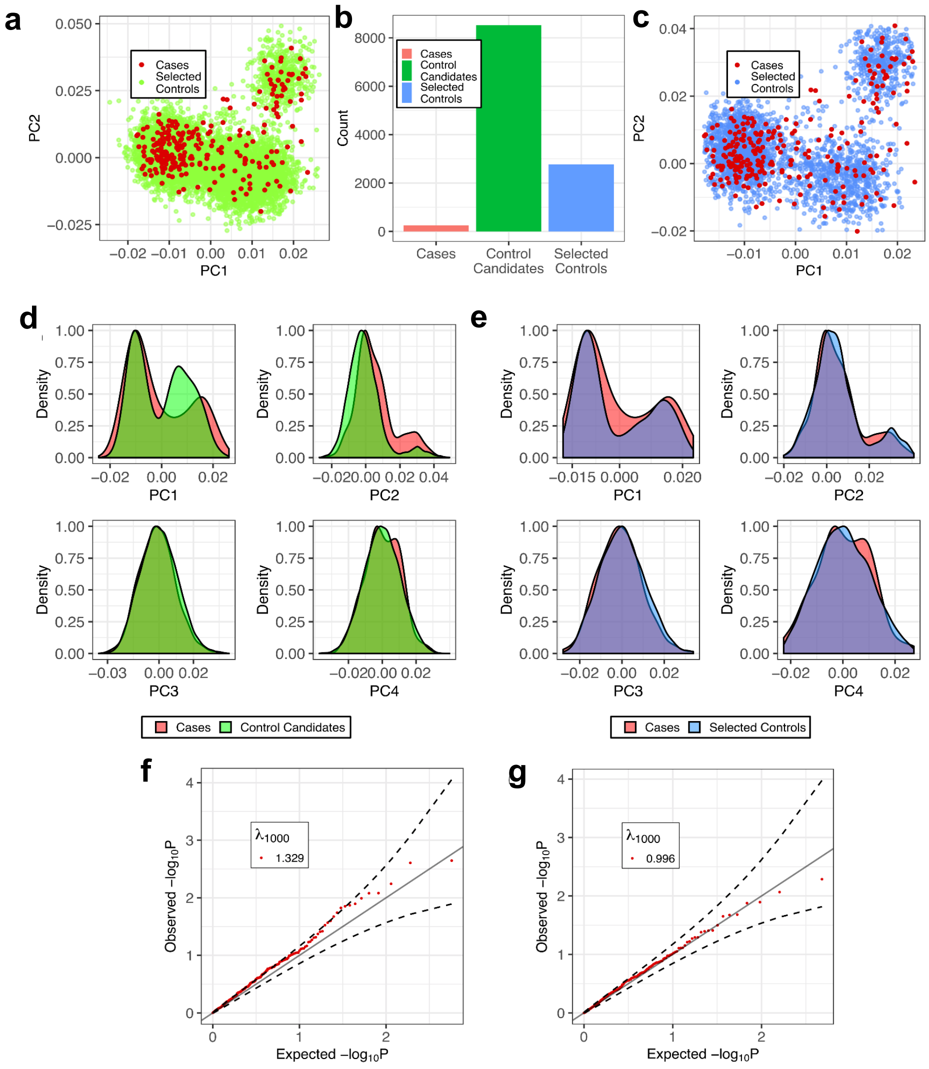

Supplementary Figure 14. Manual selection of controls from the Public Exomes dataset for the breast cancer dataset with shared genotypes.

(A) PCA for case cohort and control candidates; (B) counts of samples in each cohort; (C) PCA for case cohort and selected controls; (D) Alignment of PC distributions between cases and control candidate cohorts; (E) Alignment of PC distributions between cases and selected control cohorts; (F) QQ-plot for common synonymous variation association testing (linear regression, two-sided) in case cohort and control candidates cohort; (G) QQ-plot for common synonymous variation association testing (linear regression, two-sided) in case and selected control cohorts. Solid line represents a diagonal, dashed lines indicate 95% confidence interval.

4.4. Power calculations

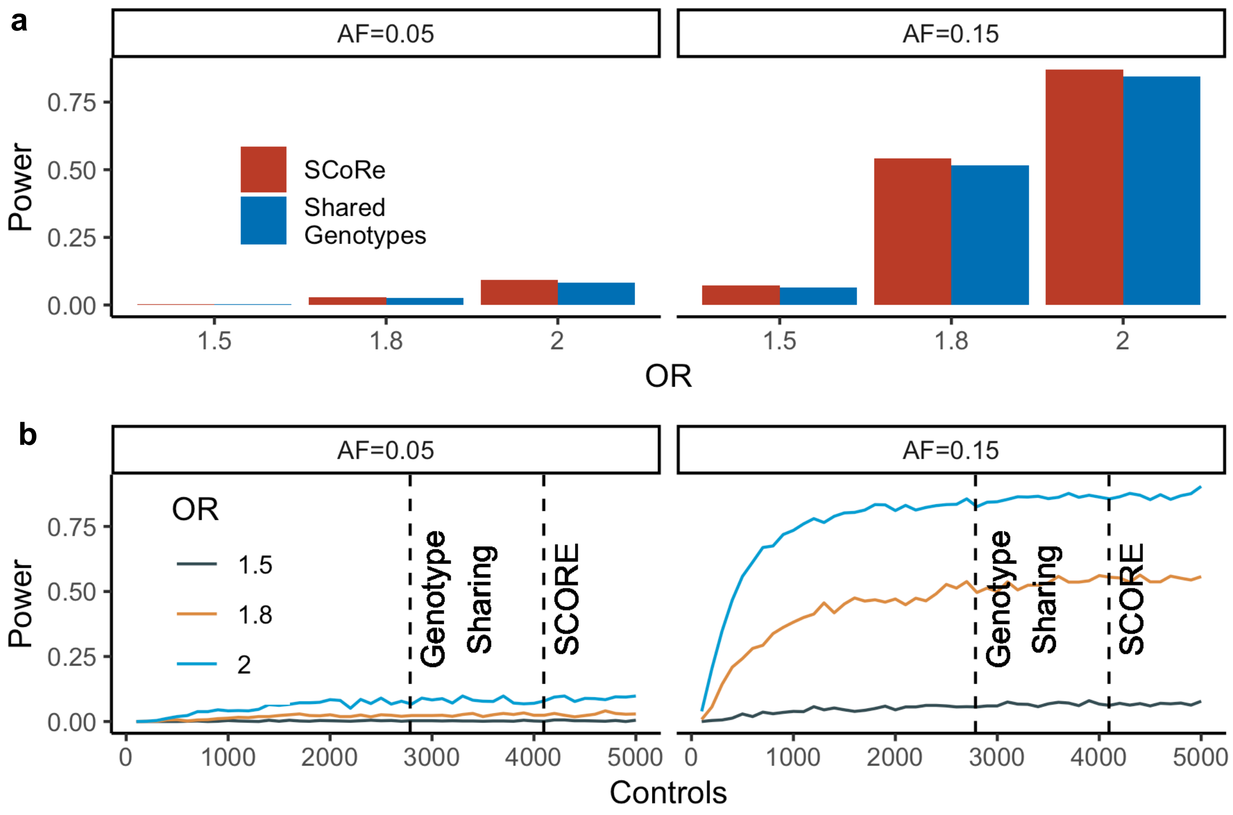

Supplementary Figure 15. BRCA power estimates. (a) Power estimates for fisher's exact test. (b) Comparison of statistical power of case-control cohort assembled by SCoRe server.

5. Case Study. TCGA African-American cohort pan-cancer association study.

We used the dataset of TCGA germline exome sequencing, assembled as described in Artomov et al[2]. Using principal component analysis, a cluster of African-American individuals, N=471. Using the *SVDFunctions* package, shareable data was prepared for *Public Exomes* control pool and controls were selected using online SCoRe platform (**Supplementary Figure 16A**). The local cohort included three ancestral clusters (**Supplementary Figure 16B**). SCoRe returned 700, 128 controls with λ=1.00, 1.03 for clusters 1 and 2, respectively. No controls within λ=0.9-1.3 were found for cluster 3. We focused downstream analyses on the largest case cluster - 1. **Supplementary Figures 16C-D** illustrate great matching quality for the variants that were used for matching and common (MAF>1%) synonymous variants that were not used in control-matching process. **Supplementary Figure 16E** shows gene-based Fisher-exact test for rare

(MAF < 1%) synonymous variants confirming adequate quality of fine-scale ancestry matching. Finally, **Supplementary Figure 16F** shows common (MAF>1%) missense and protein-truncating variants association with rs71214816 in *PRIM2* being the top-association.

We have further analyzed the clusters in the case cohort to assess the effectiveness of our approach. Cluster 1 aligned with ASW/ACB fine-scale ancestries from 1000 genomes, indicating the predominant presence of African-American descent individuals. Cluster 2 contained admixed individuals (mostly European and African-American admixture). Cluster 3 consists of the individuals which were PCA outliers and were not classified to neither cluster 1 or 2.

Selection of control cohorts for both cluster 1 and 2 indicate that our method is agnostic of the ancestry and works well for both homogenous cohorts and admixed cohorts. The only requirement that our method has is that the data can be shaped into approximately Gaussian form and we provide a specific functionality to do so.

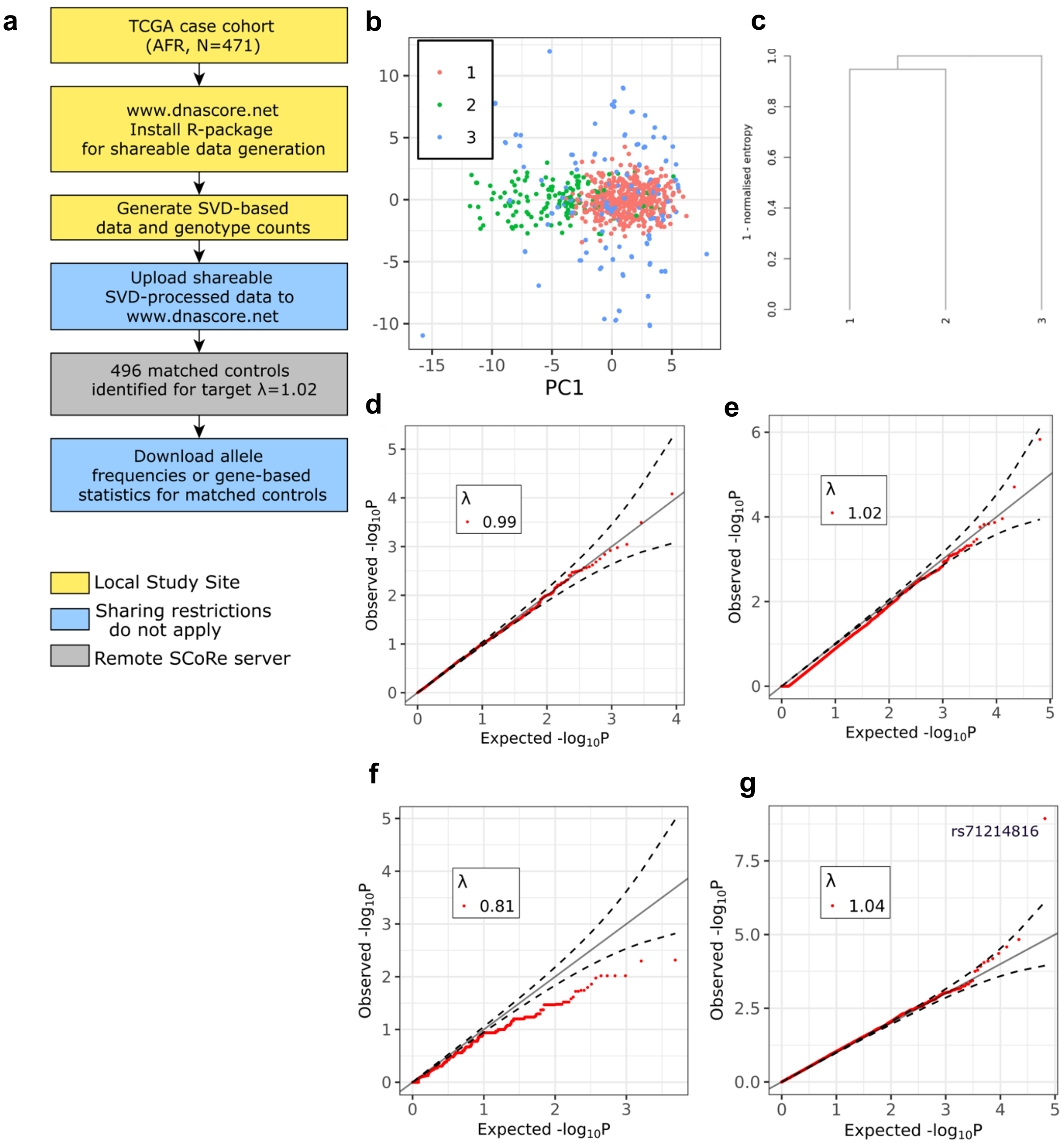

Supplementary Figure 16. Pan-cancer TCGA association study in African-American cohort without individual-level data sharing using SCoRe.

(A) Data processing scheme; (B) Clusters identified in case cohort; (C) Clustering of the case cohort; (D) QQ-plot for DNA variants used for control selection for cluster 1; (E) Common (MAF>1%) synonymous variants association for cluster 1; (F) Singleton gene-based synonymous variants association for cluster 1. Only genes with at least 5 singletons in cases shown; (G) common (MAF>1%) missense and PTV association study. In panels (D-G) solid line represents a diagonal, dashed lines indicate 95% confidence interval. Two-sided Fisher's exact test was used. Raw, unadjusted p-values reported.

As a control experiment, we then shared the genotypes between case and control cohorts and performed a conventional association study using joint PCA-based control matching, which resulted in selection of 1,706 controls for case cluster 1 and association study results that confirmed the findings made with SCoRe (**Supplementary Figure 17**).

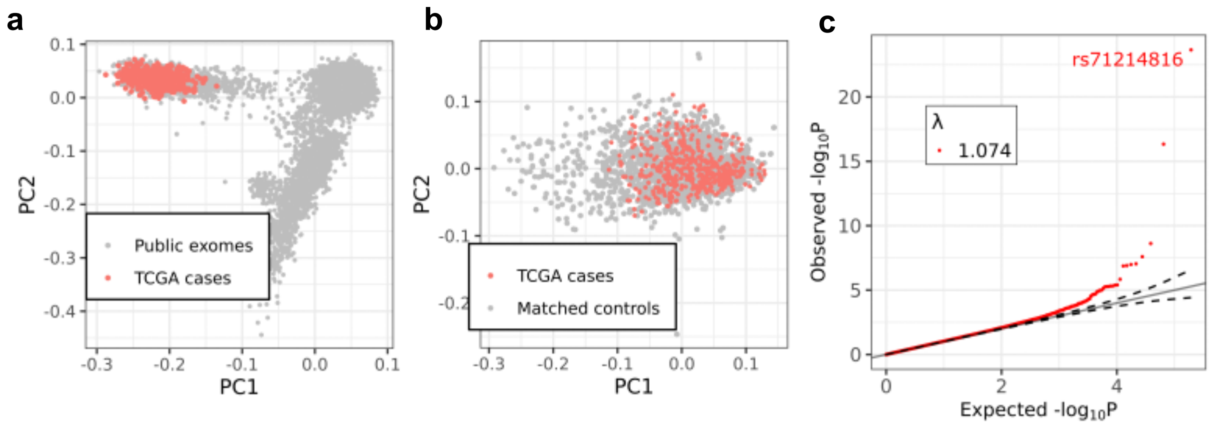

Supplementary Figure 17. Conventional genotype-sharing pan-cancer TCGA association study in African-American cohort.

(A) Joint PCA; (B) selected controls and case-cohort; (C) QQ-plot for common (MAF>1%) variants. Solid line represents a diagonal, dashed lines indicate 95% confidence interval. Two-sided Fisher's exact test was used. Raw, unadjusted p-values reported

6. Case Study. Focal Segmental Glomerulosclerosis African-American cohort association study.

We used a cohort of gene panel-sequencing focal segmental glomerulosclerosis (FSGS) assembled as described in Yu et al[3]. A cluster of African-American individuals (N=130) was identified using PCA. Importantly, the panel sequencing approach significantly limits the number of LD-independent variants that could be used for the populations structure identification, which limits the ability to use default settings of the SCoRe platform. However, it is feasible to create multiple reference basis involving variants commonly present in clinical panels to serve multiple types of data. In this case, we created a control basis space for the 724 variants that were observed in the FSGS gene panel and performed control subject selection without individual-level data sharing, consistently with the approach described for other case studies (**Supplementary Figure 18A-B**). We were able to obtain 700 controls with genomic inflation 1.00 (**Supplementary Figure 18C**). Both variants used for matching and common synonymous variants were found to be well-calibrated between case and control cohorts (**Supplementary Figure 18D**). Finally common (MAF > 5%) variants association study, replicated association of rs73885319 in *APOL1* ($p = 3.13 \times 10^{-4}$) that is a known risk factor for FSGS in African-American population (**Supplementary Figure 18D**).

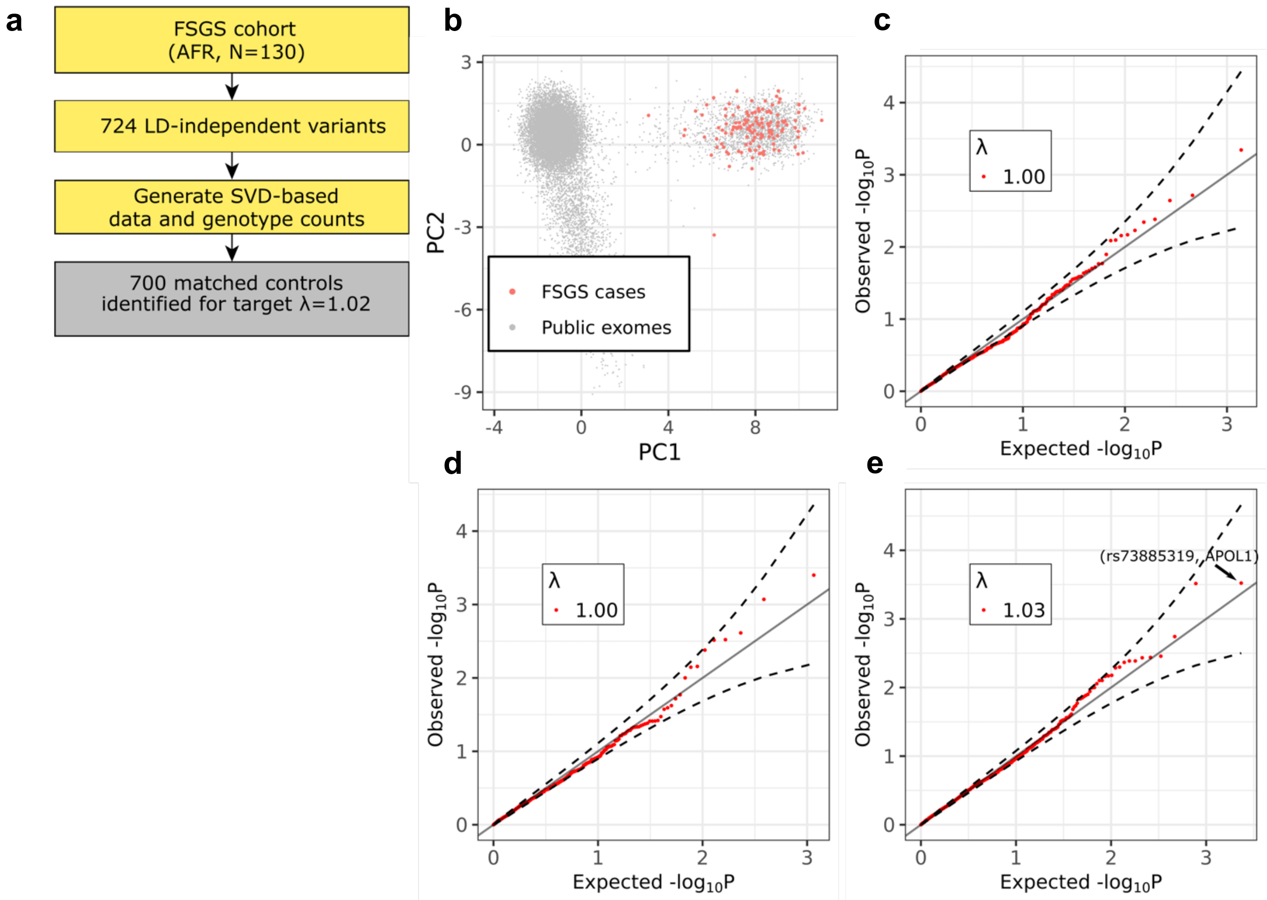

Supplementary Figure 18. Case study of FSGS in African-American population.
(A) Processing scheme for an association study without individual-level data sharing; (B) Joint PCA of FSGS cohort and control pool illustrating African-American cluster of FSGS cases; (C) QQ-plot for variants used for control selection; (D) Common (MAF>5%) synonymous QQ-plot; (E) Common (MAF>5%) missense and PTV QQ-plot showing a top association of rs73885319 in *APOL1.* In panels (C-E) solid line represents a diagonal, dashed lines indicate 95% confidence interval. Two-sided Fisher's exact test was used. Raw, unadjusted p-values reported.

7. References

1. Schubert, E. & Zimek, A. ELKI: A large open-source library for data analysis - ELKI Release 0.7.5 'Heidelberg'. (2019).
2. Artomov, M. *et al.* Case–control analysis identifies shared properties of rare germline variation in cancer predisposing genes. *Eur. J. Hum. Genet.* **27**, 824–828 (2019).
3. Yu, H. *et al.* A role for genetic susceptibility in sporadic focal segmental glomerulosclerosis. *J. Clin. Invest.* **126**, 1603–1603 (2016).
