# Supplementary material for: Public platform with 39,472 exome control samples enables association studies without genotype sharing
Source: Nat Genet. 2024 Jan 10;56(2):327–35. doi: 10.1038/s41588-023-01637-y (PMC10864173; doi:10.1038/s41588-023-01637-y)
Supplement: Supplementary file 2 — Reporting Summary [file 41588_2023_1637_MOESM2_ESM.pdf]

nature portfolio

Corresponding author(s): Mykyta Artomov

Last updated by author(s): Nov 1, 2023

Reporting Summary

Nature Portfolio wishes to improve the reproducibility of the work that we publish. This form provides structure for consistency and transparency in reporting. For further information on Nature Portfolio policies, see our Editorial Policies and the Editorial Policy Checklist.

Statistics

For all statistical analyses, confirm that the following items are present in the figure legend, table legend, main text, or Methods section.

n/a	Confirmed	
☐	☒	The exact sample size (*n*) for each experimental group/condition, given as a discrete number and unit of measurement
☐	☒	A statement on whether measurements were taken from distinct samples or whether the same sample was measured repeatedly
☐	☒	The statistical test(s) used AND whether they are one- or two-sided<br>*Only common tests should be described solely by name; describe more complex techniques in the Methods section.*
☐	☒	A description of all covariates tested
☐	☒	A description of any assumptions or corrections, such as tests of normality and adjustment for multiple comparisons
☐	☒	A full description of the statistical parameters including central tendency (e.g. means) or other basic estimates (e.g. regression coefficient) AND variation (e.g. standard deviation) or associated estimates of uncertainty (e.g. confidence intervals)
☐	☒	For null hypothesis testing, the test statistic (e.g. *F*, *t*, *r*) with confidence intervals, effect sizes, degrees of freedom and *P* value noted<br>*Give P values as exact values whenever suitable.*
☒	☐	For Bayesian analysis, information on the choice of priors and Markov chain Monte Carlo settings
☐	☒	For hierarchical and complex designs, identification of the appropriate level for tests and full reporting of outcomes
☐	☒	Estimates of effect sizes (e.g. Cohen's *d*, Pearson's *r*), indicating how they were calculated

Our web collection on statistics for biologists contains articles on many of the points above.

Software and code

Policy information about availability of computer code

Data collection	No specific software was used for data collection
Data analysis	R-package SVDFunctions was developed to be used complementary to the control repository at www.dnascore.net.<br>All code is freely available at https://github.com/alexloboda/SVDFunctions (doi: 10.5281/zenodo.6778054) and a tutorial is available at www.dnascore.net.<br>GATK, BWA and Picard Tools were used for control datasets creation and preprocessing.

For manuscripts utilizing custom algorithms or software that are central to the research but not yet described in published literature, software must be made available to editors and reviewers. We strongly encourage code deposition in a community repository (e.g. GitHub). See the Nature Portfolio guidelines for submitting code & software for further information.

Data

Policy information about availability of data

All manuscripts must include a data availability statement. This statement should provide the following information, where applicable:

- Accession codes, unique identifiers, or web links for publicly available datasets
- A description of any restrictions on data availability
- For clinical datasets or third party data, please ensure that the statement adheres to our policy

No new data was generated in this work. All datasets used for creation of the control repository could be obtained from the dbGAP or from the dedicated

repository. Complete list of links is available in Supplementary Tables 1 and 2. SCoRe control repository could be accessed at http://dnascore.net . Tutorial and instructions on how to use the package and repository are provided at the "Tutorial" tab of the SCoRe web-site. The following public (available through dbGAP) datasets were used for creation of the control database: https://www.internationalgenome.org/data/, phs000814.v1.p1
(dbGAP), http://evs.gs.washington.edu/EVS/, phs000806.v1.p1
(dbGAP)
, phs001552.v1.p1 (dbGAP), https://www.ncbi.nlm.nih.gov/pubmed/29165699 , https://thl.fi/en/web/thl-biobank/for-researchers/sample-collections
, https://thl.fi/en/web/thl-biobank/for-researchers/sample-collections , https://thl.fi/en/web/thl-biobank/for-researchers/sample-collections , https://www.ncbi.nlm.nih.gov/pubmed/29165699
https://thl.fi/en/web/thl-biobank/for-researchers/sample-collections
, http://www.type2diabetesgenetics.org/projects/t2dGenes
http://www.type2diabetesgenetics.org/projects/t2dGenes
http://www.type2diabetesgenetics.org/projects/t2dGenes
http://www.type2diabetesgenetics.org/projects/t2dGenes
https://www.uk10k.org/data_access.html
Breast cancer cohort is available at dbGAP through phs000822.v1.p1.
TCGA cohort is available at dbGAP through phs000178.v11.p8.

Human research participants

Policy information about studies involving human research participants and Sex and Gender in Research.

Reporting on sex and gender	N/A
Population characteristics	We provide access to two large-scale sequencing datasets - Public exomes, which includes major continental populations and Nordic dataset, which includes Finnish and Swedish origin participants
Recruitment	N/A
Ethics oversight	Non-human subject research was determined for the project, as no identifiable data was used

Note that full information on the approval of the study protocol must also be provided in the manuscript.

Field-specific reporting

Please select the one below that is the best fit for your research. If you are not sure, read the appropriate sections before making your selection.

☒ Life sciences ☐ Behavioural & social sciences ☐ Ecological, evolutionary & environmental sciences

For a reference copy of the document with all sections, see nature.com/documents/nr-reporting-summary-flat.pdf

Life sciences study design

All studies must disclose on these points even when the disclosure is negative.

Sample size	We assembled largest exome sequencing studies available through dbGAP into a single dataset and utilized the vast majority of all Finnish and Swedish exome sequencing data available to date.
Data exclusions	Call rate filters were applied on individual and variant levels to exclude poor quality sequencing data
Replication	The methodology for control selection without genotype sharing was applied in different independent settings: multiple continental populations, multiple local populations, different genotype discovery technologies (microarray, exome sequencing, panel sequencing) and different exome sequencing platforms (Agilent and Nextera).
Randomization	We used cross-validation (random sampling of the dataset) to simulate case cohorts from a given continental ancestry. At least 10 rounds of random sampling were conducted in every setting.
Blinding	To test our control matching and selection algorithm no genotype or other individual-level data was used when interacting with control database to select controls. The SCoRe server was tested in a blinded way, without any assumptions provided to the control server.

Reporting for specific materials, systems and methods

We require information from authors about some types of materials, experimental systems and methods used in many studies. Here, indicate whether each material, system or method listed is relevant to your study. If you are not sure if a list item applies to your research, read the appropriate section before selecting a response.

Materials & experimental systems

n/a	Involved in the study
☒	☐ Antibodies
☒	☐ Eukaryotic cell lines
☒	☐ Palaeontology and archaeology
☒	☐ Animals and other organisms
☒	☐ Clinical data
☒	☐ Dual use research of concern

Methods

n/a	Involved in the study
☒	☐ ChIP-seq
☒	☐ Flow cytometry
☒	☐ MRI-based neuroimaging
